# Porous Structural Microfluidic Device for Biomedical Diagnosis: A Review

**DOI:** 10.3390/mi14030547

**Published:** 2023-02-26

**Authors:** Luyao Chen, Xin Guo, Xidi Sun, Shuming Zhang, Jing Wu, Huiwen Yu, Tongju Zhang, Wen Cheng, Yi Shi, Lijia Pan

**Affiliations:** Collaborative Innovation Center of Advanced Microstructures, School of Electronic Science and Engineering, Nanjing University, Nanjing 210093, China

**Keywords:** microfluidic, porous structure, biomedical, biosensor

## Abstract

Microfluidics has recently received more and more attention in applications such as biomedical, chemical and medicine. With the development of microelectronics technology as well as material science in recent years, microfluidic devices have made great progress. Porous structures as a discontinuous medium in which the special flow phenomena of fluids lead to their potential and special applications in microfluidics offer a unique way to develop completely new microfluidic chips. In this article, we firstly introduce the fabrication methods for porous structures of different materials. Then, the physical effects of microfluid flow in porous media and their related physical models are discussed. Finally, the state-of-the-art porous microfluidic chips and their applications in biomedicine are summarized, and we present the current problems and future directions in this field.

## 1. Introduction

Microfluidics, also known as lab-on-a chip (LOC), is a device that integrates biomedical analysis experiments, including sample preparation, separation, reaction, detection, etc. [1,2,3]. Microfluidic chips can achieve rapid results with a small amount of samples through the precise control of fluids. Since scientists developed micron-scale gas chromatography columns on silicon chips in 1979, microfluidic chips have undergone considerable development and have been widely used in the fields of biology, chemistry and medicine [4,5,6,7,8,9,10,11]. At present, microfluidic chips are still facing challenges such as large interference and difficult separation. Therefore, various innovative technologies based on preparation methods and structure have been proposed to achieve better performance. For example, the combination of intelligent communication devices and microfluidic chips has been proposed to enable easier real-time healthcare detection [12,13,14,15]. The combination of spinning technology and microfluidic chips is applied to flexible wearable devices [16,17,18,19,20].

In addition to device preparation methods and structural innovations, changing the structure of materials can also play a role in improving the sensing performance, stability and sensitivity of microfluidic devices. Porous materials, with their high specific surface area, good permeability, low relative density, and high specific strength, are widely used in microfluidic chips for applications such as cell separation, cell culture, and real-time biomedical detection [2,21,22,23,24]. Due to its high specific surface area, the porous structure provides a basis for achieving a higher capture rate and provides another idea for improving detection sensitivity. The original and most widely used porous structure material is paper. As a material with a natural porous structure, paper has the advantages of low cost and light weight. In 1952, Martin and Synge invented paper-based chromatographic separation technology and won the Nobel Prize in Chemistry [25]. Since then, paper-based microfluidic devices have been widely used in detection and medical treatment, and there are many related literatures reviewing their related applications [1,8,26]. In addition, with the development of microelectronics technology in recent years, another porous material commonly used in the field of biomedical detection is polymers such as PDMS. Compared with traditional glass and silicon materials, PDMS has the advantages of elasticity, castability, and surface chemical properties that can be modified, and it is increasingly used in microfluidic devices [27,28,29,30]. Multi-well PDMS screening plates are commonly used in cell sorting and cell culture. There are also novel flexible porous materials such as hydrogels and textile fabrics for microfluidic applications, which we will describe in detail later in the article.

**Figure 1:**
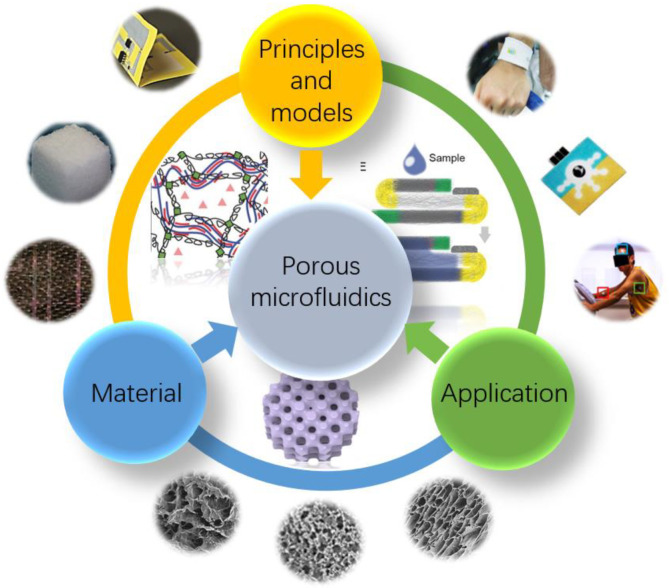
Illustration of the structure of this review. With the help of advanced material and theoretical modeling, the constantly advancing applications of porous microfluidic device for biomedical diagnosis. Reproduced with permission. Ref. [31] Copyright 2012, Sensors and Actuators B: Chemical. Reproduced with permission. Ref. [32] Copyright 2017, Advanced Functional Materials. Reproduced with permission. Ref. [33] Copyright 2017, Angewandte Chemie International Edition. Reproduced with permission. Ref. [34] Copyright 2019, Science Advances. Reproduced with permission. Ref. [35] Copyright 2011, ACS Appl Mater Interfaces. Reproduced with permission. Ref. [36] Copyright 2019, Acta Biomater. Reproduced with permission. Ref. [37] Copyright 2016, Adv Mater. Reproduced with permission. Ref. [38] Copyright 2016, Adv Mater. Reproduced with permission. Ref. [39] Copyright 2012, ACS Appl Mater Interfaces. Reproduced with permission. Copyright 2014, Anal Chem [40].

In this review, various microfluidic devices based on porous structure will be discussed by material, including their characteristics and fabrication methods. In order to better understand the flow mechanism of fluids in porous structured microfluidic channels, in the next section, relevant physical principles and the fluid dynamics related models of porous structures are discussed. Then, the application of microfluidic devices based on porous structures in biomedical diagnostics will be discussed in scenarios (Figure 1). Finally, we will summarize and look forward to the future development of porous microfluidic devices.

## 2. Different Materials and Preparation Methods for Microfluidic Devices Based on Porous Structures

The most outstanding feature of the microfluidic system is its ability to analyze and manipulate microliters of fluid in channels with diameters of 10–100 μm [41,42]. A porous structured microfluidic system with small thermal mass, high mass transfer efficiency, high specific surface area, good permeability, low relative density and high specific strength can effectively improve the flux rate and sensitivity of the device [43,44,45,46,47,48,49]. The diversity of porous materials stems from a wide range of preparation methods [50,51,52,53,54]. Because of their controllable pore size and porosity, porous materials offer a wide range of applications for microfluidic systems [9,55,56,57,58,59].

Initially, with the development of the silicon-based industry, the first generation of microfluidic chips was created and prepared on silicon/silicon dioxide substrates by traditional microfabrication methods [60,61]. The typical microfabrication process consists of three steps: lithography and development, etching to form microchannels, and bonding to assemble the microfluidic chip. Silicon-based microfluidic chips were first developed and have advantages such as high thermal conductivity, special optical properties and electro-osmotic stability. However, their high cost, complex process flow, time consuming nature, rigidity and gas impermeability and other shortcomings have become obstacles to the large-scale application of health monitoring and wearable biomedicine [10,60,62,63,64].

To meet the market demand for the mass production of microfluidics for biomedical and clinical applications, chips are required to be flexible, breathable, low cost, disposable, and environmentally friendly. Therefore, microfluidic devices based on porous three-dimensional structures of paper, soft elastic materials and textile fabrics have been created [1,37,65,66].

### 2.1. Fabrication of PDMS and PMMA-Based Microfluidics

Synthetic polymers are ubiquitous materials used in a wide variety of applications because of their structural and mechanical properties [67]. The application of polymers as a discontinuous dielectric structure in wearable devices offers a wide range of applications in health monitoring and medical fields [27,68,69,70]. The rapid development of porous polymers in the last few years has been attributed to the continuous development of modern organic synthesis, advanced polymerization techniques and nanotechnology [71].

Polydimethylsiloxane (PDMS) has been widely used in many research fields because of its excellent properties such as easy fabrication, high flexibility, and thermal stability [72]. PDMS is a commonly used material in microfluidics. The layer-by-layer process allows the fabrication of 3D microfluidic molds for casting PDMS. Two-dimensional (2D) PDMS layers can be combined together to form 3D structures. Techniques such as the sacrificial template method and soft lithography are also commonly applied to the preparation of PDMS materials with porous structures [29,73,74]. UV modifications are also frequently used on PDMS to modify the surface properties by tuning their intensity [75,76]. These modifications alter the wettability by adding functional groups, creating thin layers on the PDMS surface and significantly enhancing the potential for microfluidic applications.

**Figure 2:**
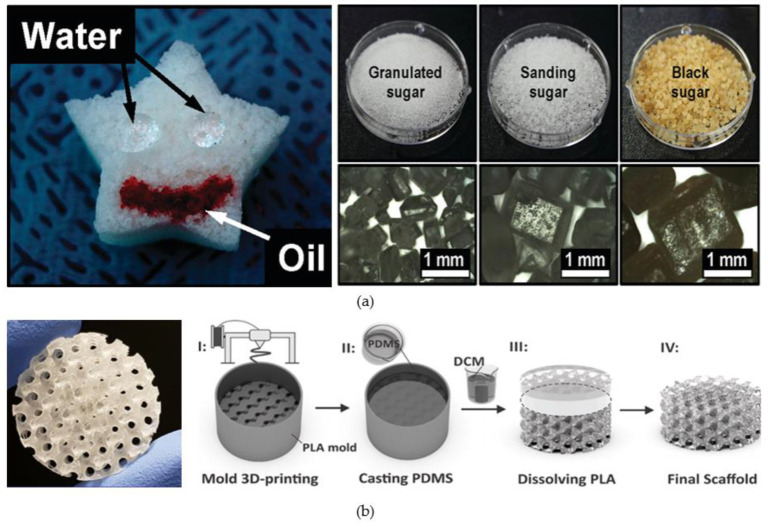
(**a**) Porous PDMS sponges and optical microscope images of various sugar particles; (**b**) Porous PDMS scaffolds and its fabrication process. (**a**) Reproduced with permission. Ref. [35] Copyright 2011, ACS Appl Mater Interfaces. (**b**) Reproduced with permission. Ref. [36] Copyright 2019, Acta Biomater.

Due to the simplicity, safety and low cost of preparation, the sacrificial template method is commonly used to obtain porous PDMS sponges [36,77]. The successful fabrication of porous PDMS using sugar as a template and water as a solvent was first reported by Choi et al. [35]. PDMS sponges composed of porous, interconnected three-dimensional frameworks were prepared by using cubic sugars of different particle sizes as templates (Figure 2a). This structure of PDMS sponge can largely bend and recover the original shape almost perfectly, thus greatly improving the stability of flexible microfluidic devices. Similarly, Zhao et al. prepared porous PDMS sponges using NaCl instead of cubose as a template [78]. Yu et al. used citric acid monohydrate (CAM) as a template and ethanol as a solvent to make 3D interconnected porous PDMS sponges with high porosity, flexibility and superwettability [72]. Other early conventional methods for preparing porous microfluidic chips include photolithography and etching techniques [79,80,81,82]. However, the dies fabricated by complex processes such as photolithography are limited by flexibility, and the die pattern cannot be changed once it is fabricated. At the same time, the traditional photolithography and etching methods have the disadvantages of high cost, complicated process and being time consuming. In contrast, laser micromachining and printing technology becomes a better choice because of its simplicity, flexibility, controlled aperture size, speed and direct writing of different collection shapes [83,84,85]. Chen et al. report on printing protocols for PDMS with porous structures printed using liquid dispensers, flow control capabilities, flow delay mechanisms, and applications in sequential delivery [30]. Montazerian et al. prepared a PDMS porous scaffold with triply periodic minimal surfaces structure with radial gradient porosity by combining 3D printing technology [36]. This porous structure of PDMS material is highly elastic, water permeable and biocompatible (Figure 2b).

Another material commonly used to prepare microfluidic devices is PMMA, which is a thermoplastic material that has better mechanical properties than PDMS and can maintain good initial morphology under mechanical stress conditions. In addition, PMMA has the advantages of stable chemical properties, optical transparency and low cost. The common methods used to process PMMA are hot embossing, injection molding and direct laser writing. Bouchard et al. fabricated periodic patterns with linear and dotted geometrical features on stainless steel surfaces with sizes ranging from 1.7 to 900 µm by combining different laser-based processes, namely direct laser engraving (DLE), direct laser writing (DLW), and direct laser interference patterning (DLIP). Then, the fabricated layered geometries are transferred to the PMMA surface by plate-to-plate thermal embossing [86]. Volpe et al. developed and tested a new intelligent procedure for the rapid fabrication of PMMA LOC prototypes for cell capture by using femtosecond laser technology for microchannel mechanical micro-milling of the inlet and outlet connections and thermal bonding to complete the device [87]. The snake microchannels are then directly biofunctionalized by fixing capture probes, which can distinguish between cancer and non-cancer cells without labeling. The device is useful for the label-free capture and identification of tumor cells from blood cells.

As the traditional materials for fabrication microfluidic devices, PDMS and PMMA play an important role in microfluidics due to their simple processing (compared to silicon and glass), excellent mechanical properties and low preparation cost, and they will have a broader commercial product and market demand in the future.

### 2.2. Fabrication of Paper-Based Microfluidics

Paper has unique advantages over other materials in terms of low cost, flexibility and self-driven fluid pumping, thus making it widely used in various fields of wearable devices and human health monitoring [88,89,90,91,92]. Paper-based microfluidics is a fast-growing field because the inherently porous structure of paper provides a separate site for transporting liquids by capillary forces [1,93,94,95]. In recent years, with the rapid development in the field of materials, the definition between traditional paper and flexible films has become blurred, and some flexible films with flexible or porous structures have been defined as paper [96]. Some treatment and processing of the microstructure of the paper is usually necessary to create paper-based microfluidic devices, including wax printing, printing, photolithography, etching, chemical alteration of the fiber surface, and other methods [97,98,99,100].

**Figure 3:**
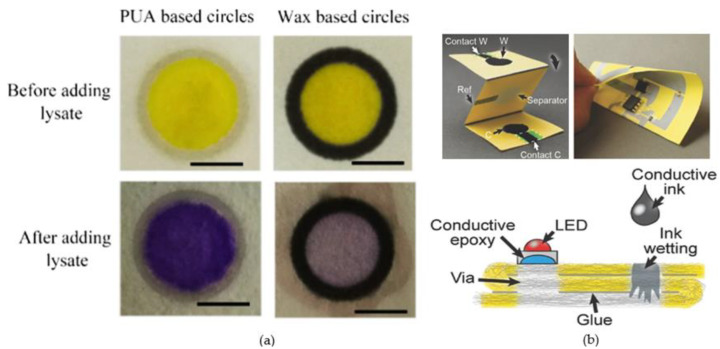
(**a**) Microfluidic paper-based analytical devices for colorimetric assays for *E. coli* BL21; (**b**) Photos and schematic diagram of the paper-based printed 3D circuit integrating electronics and microfluidics. (**a**) Reproduced with permission. Ref. [101] Copyright 2020, Sensors and Actuators B: Chemical. (**b**) Re-produced with permission. Ref. [37] Copyright 2016, Adv Mater.

Photolithography was used to create the first paper-based microfluidic devices and is still the most common preparation method due to its advantages such as high resolution and accuracy [3]. Photolithography begins by covering the paper with photoresist and forming a proper patterned cross-linked photoresist by using a photomask. Finally, the remaining photoresist is eliminated to form patterned microstructures. Dong et al. proposed a microfluidic paper-based analytical device fabricated by UV curing using aqueous polyurethane acrylate (PUA) to pattern the filter paper [101] (Figure 3a). Wax is a non-toxic, disposable, low-cost and easily patterned hydrophobic substance. Therefore wax printing has become a popular process for preparing disposable paper-based microfluidic devices [100,102,103]. A sheet of paper with a wax print is heated to allow the wax to flow and allow it to reach the thickness of the paper. This creates a completely impermeable hydrophobic barrier with a hydrophilic zone inside it shaped like a wax print pattern. Carrilhoo et al. elaborate on the wax-printing technique, where a roll of paper is first passed through a wax printer, then heated through an oven, and finally through an inkjet printer, where reagents for testing or other applications are printed in the test area [104]. Mani et al. prepared low-cost paper-based microfluidic devices for patterning using a wax-printing method. The microfluidic structures were made by simply patterning them. Equipping the device with RuPVP/DNA/enzyme spots can be used to demonstrate screening for genotoxic compounds in water, food and smoke [105]. The integration of printed electronics and microfluidics on paper is still in the early stages of development. Hamedi et al. added ink to the microchannels in combination with electronic printing techniques to form internal paper conductors. The ink covered the cellulose fibers without clogging the pores of the paper and kept the channels hydrophilic [37] (Figure 3b). Although devices for paper-based microfluidic devices have experienced rapid development in recent years, there are still many key challenges to overcome such as resolution, biocompatibility and precision.

### 2.3. Fabrication of Three-Dimensional Hydrogels and Textile Fabrics-Based Microfluidics

**Figure 4:**
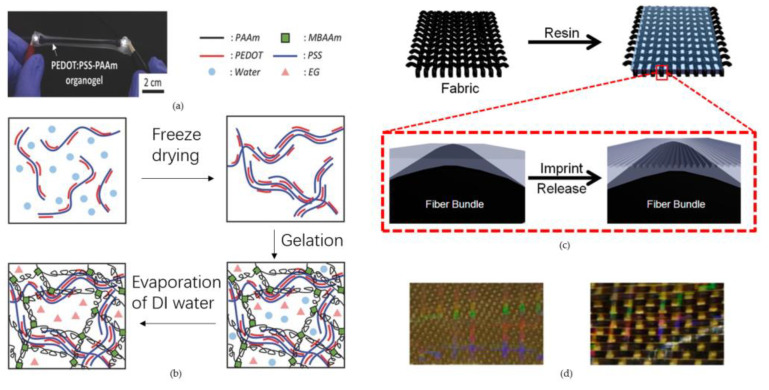
(**a**) Photo of PETDOT:PSS-PAAm organogel stretch up to 200% strain; (**b**) Synthesis procedure of the electrically conductive PEDOT:PSS–PAAm organogels; (**c**) Schematic of the fabrication of a patterned fiber composite; (**d**) Macroscopic photographs of E-glass/x-PDMS and Kevlar-carbon fiber/x-PDMS. (**a**,**b**) Reproduced with permission. Ref. [38] Copyright 2016, Adv Mater. (**c**,**d**) Reproduced with permission. Ref. [39] Copyright 2012, ACS Appl Mater Interfaces.

Although significant progress has been made in the past few years in the fabrication of PDMS flexibles, 2D papers and membranes, they still suffer from the drawbacks of limited biocompatibility and poor mechanical properties, which still hinder the wide application of microfluidic devices in biomedical applications. Three-dimensional cross-linked networks combined with conductive hydrogels and three-dimensional electrotextiles have received widespread attention in the field of wearable microfluidic devices using their unique tunable mechanical flexibility, high biocompatibility and excellent electronic properties [106,107]. The current preparation of conductive hydrogels is generally based on co-networking, self-assembly and blend techniques [108,109,110,111]. Joo et al. prepared conductive hydrogels composed of poly(3,4-ethylenedioxythiophene), in which polystyrene sulfonate (PEDOT:PSS) acted as the main conductive pathway for electrical signal transmission and the hydrogel cross-linked polymer network was highly stretchable [38] (Figure 4a). Some ions are added to the hydrogel, which increases the electrical conductivity of the conductive hydrogel by increasing ion migration due to the presence of large amounts of water in the hydrogel network (Figure 4b). Zhi et al. developed cross-linked polyacrylamide hydrogels with electrical conductivity provided by vinyl hybridized silica nanoparticles [33]. Crosby et al. proposed a simple one-step embossed lithographic patterning method to form flexible and highly stretchable patterned composite fabrics by an embossed photolithography procedure [39] (Figure 4c). By regulating the arrangement of the fibers and the three-dimensional shape of the fabric, the anisotropy of the material will be regulated (Figure 4d). This property of the fabric maintains a higher degree of directional bending flexibility than conventional flexible materials. Chen and colleagues synthesized porous graphene fibers by microfluidic orientation strategy [32]. The fibers have a uniformly dense porous network, excellent flexibility and superior electrical conductivity, providing excellent applications for wearable medical electronics.

Table 1 summarizes the above-mentioned methods for the preparation of porous structures of different materials and provides some additional information. For each method, we have listed the advantages and limitations, and readers can choose the appropriate method according to their need in different scenarios.

## 3. Relevant Principles in Microchannels and Hydrodynamically Relevant Models for Porous Structures

Understanding the flow behavior of microfluids in porous structures has important implications not only for theory but also for practical applications. Usually, the dynamics of microfluids obeys the linear Stokes equations, but due to the existence of porous structures, the variable interfacial tension and specific boundary conditions change to produce nonlinear behavior [60,136,137,138]. Fluid dynamics can only provide some guidance at this point, while the reality of the problem becomes more complex. Therefore, some complex real-world problems can be solved by some flexible application platforms and simulations.

### 3.1. Inertial Effect of Microfluidics

From the above, it can be seen that the flow of microfluidic devices can be considered as Stokes flow when the Reynolds number of the flow field is neglected [139]. When inertia and viscosity effects are taken into account, the particle flow will no longer conform to the Stokes equations, thus becoming more complicated [140]. The inertial effect was first discovered in macroscopic pipes, where initially millimeter-sized suspended particles randomly distributed in a circular pipe (about 1 cm) migrated laterally to focus on a ring with a radius 0.6 times the radius from the center of the pipe [141]. Under moderate Reynolds number conditions, the migrating ions in the circular channels form the Segre–Silberberg annulus due to the symmetry of the rings [142]. In a curved channel, where the fluid in the central region has a higher velocity than in the wall region, the Poiseuille flow has a velocity curve similar to a parabola in the main flow direction. When the particles pass through the curved closed channel, in order to satisfy the mass conservation, the fluid near the outer wall is recirculated inward due to the centrifugal pressure gradient, and two vortices with opposite rotational directions are generated in the cross-sectional direction (lab on a chip) (Figure 4). This fluid motion profile for cross-sectional quadratic flow was first proposed by Dean and is characterized by the dimensionless coefficient De:

(1) $$D_{e}=R_{e}\sqrt{\frac{H}{2R}}$$

where R is the curvature radius of the channel, and H is the hydraulic diameter [143,144]. Dinler et al. simulated the fluid motion behavior of particles under the action of the Dean vortex and predicted the focusing bandwidth for particles of different sizes [145]. Due to the presence of secondary flow, the particles in the curved channel are also affected by the Dean drag force: FD∝ρU2apH2R−1, where U is the velocity of the fluid, H is the hydraulic diameter, ap is the diameter of the particle, ρ is the density of the fluid, and R is the curvature radius of the channel. Gossett believes that at low velocities, the particles are subject to the interaction of transverse inertial lift and the Dean drag force [146].

### 3.2. Electrorheological Effect of Microfluidics

When the fluid and the particles dispersed in the fluid are non-conductive or slightly conductive, an applied electric field is applied to the dispersion and the particles will be electrodepolarized due to the different dielectric constants of the objects. The induced dipole moment can be expressed as:

(2) $$\vec{P}=\frac{\varepsilon_{s}-\varepsilon_{\iota }}{\varepsilon_{S}+2\varepsilon_{\iota }}R^{3}\vec{E_{l}}={\beta R}^{3}\vec{E_{l}}$$

where εs and εl denote the complex permittivity of solid particles and liquids, respectively, and R denotes the radius of the sphere, β is the Claussius–Mossotti (CM) factor, and El is the field at the local field. The particles will tend to aggregate and form chains along the direction of the applied field, which is a phenomenon that is the increasing viscosity behavior exhibited by colloids when sheared in the direction perpendicular to the electric field [147]. Earliest, the model of the induced dipole–dipole interaction proposes that for the same system of dielectric microspheres dispersed in an insulating fluid, the lowest energy state is where the microspheres aggregate and form columns along the applied field direction, and the microstructure inside the columns can be predicted from the dipole–dipole interactions [148]. In recent years, Yethiraj et al. discovered the phase behavior of colloidal systems with tunable interparticle interactions under an external electric field by confocal microscopy [149]. Hynninen et al. used simulations to calculate the Helmholtz free energy of a similar system [150], and the simulation results obtained were in good agreement with the experimental results of Yethiraj et al. Although many problems of dielectric electrorheological fluids can be obtained and solved from simulations and experiments, they cannot provide an overview of dielectric electrorheological mechanisms. The microstructural changes in the rheological properties induced by the electric field should also be accompanied by electrical manifestations. In the presence of an electric field, the effective dielectric constant of the system reflects anisotropy:

(3) $$\langle \vec{D}\rangle ={\bar{\varepsilon }}_{\mathrm{eff}}\langle \vec{E}\rangle$$

(4) $${\bar{\varepsilon }}_{\mathrm{eff}}=\left(\begin{array}{ccc} {\bar{\varepsilon }}_{\mathrm{xx}} & {\bar{\varepsilon }}_{\mathrm{xy}} & {\bar{\varepsilon }}_{\mathrm{xz}}\\ {\bar{\varepsilon }}_{\mathrm{yx}} & {\bar{\varepsilon }}_{\mathrm{yy}} & {\bar{\varepsilon }}_{\mathrm{yz}}\\ {\bar{\varepsilon }}_{\mathrm{zx}} & {\bar{\varepsilon }}_{\mathrm{zy}} & {\bar{\varepsilon }}_{\mathrm{zz}} \end{array}\right)$$

The concept of an effective dielectric constant is based on the nature of the interaction of electromagnetic waves with non-uniform materials. When the wavelength is large, the microstructure cannot be resolved, and the composite material appears homogeneous to the probe wave, whose electromagnetic response is completely captured by the effective permittivity tensor [151]. The Gibbs free energy density of the fluid–solid complex with the participation of the dielectric constant tensor is:

(5) f=−18πE→⋅Reε¯eff⋅E→−TS=−18πReε¯zzE2−TS

where S is the entropy and Reε¯zz denotes the real part in parentheses. The method of variable effective permittivity allows us to obtain the dielectric electrorheological ground state structure, thus providing a basis for quantitative assessment of the rheological properties that may arise from structural deformation [152,153].

### 3.3. Fluid Behavior in Porous Media

Paper has the advantages of being inexpensive, being easy to obtain and having a high specific surface area, making it the most common medium in porous [26,37]. Paper consists essentially of a random distribution of cellulose, and the flow of liquid in paper is dominated by capillary [1,154]. The Lucas–Washburn (L-W) model considers the porous system as a bundle of parallel rigid capillaries with an average cross-section. Its equation is expressed as:

(6) $$x\left(t\right)=\sqrt{\frac{r\sigma t\;\cos\theta }{2\mu }}$$

where σ is the surface tension (gas–liquid), μ is the dynamic viscosity of the liquid, r is the capillary radius, and θ is the contact angle between the capillary wall and the liquid [155]. When used to describe the fully saturated wetting flow of a liquid through a paper system, it can only be used in the case of one-dimensional flow of a single homogeneous porous system. Darcy’s model was used to capture capillary-driven transport through porous structures in a multidimensional flow case [156]. The volumetric flow rate through a porous paper medium under this model is:

(7) $$Q=-\frac{\mathrm{kA}}{\mu x}\Delta P$$

where k is the permeability, μ is the dynamic viscosity of the fluid, A is the cross-sectional area, and ΔP is the pressure difference across the length x [157]. However, both models are based on the assumption of full saturation by the wetting front of the liquid. Buser et al. proposed a model for flow in partially saturated porous paper media by means of the Richards equation:

(8) $$\frac{\delta \theta }{\delta t}=\frac{\delta }{\delta z}\left[K\left(\theta \right)\frac{\delta H\left(\theta \right)}{\delta z}\right]$$

where K is the hydraulic conductivity and H is the pressure head; the equation relates the change in saturation of the porous medium to the pressure head gradient [158]. This model can be thought to accurately describe the fluid flow in porous media.

Early theoretical analysis of flow in porous media used a coarse-grained approach, which employs macroscopic properties. For Newtonian flow, Darcy et al. proposed a relationship between the pressure drop per unit length ΔP/L and the average velocity V:

(9) $$\frac{\Delta P}{L}=\frac{\eta V}{K}$$

where η is the viscosity of the fluid and the permeability K is a constant that depends on the properties of the medium [159]. However, this equation is limited to describe simple flows and Newtonian flows on a macroscopic scale. For the flow of a fluid through a porous structure, the classical problem of flow around a cylinder can be a benchmark for non-Newtonian fluid dynamics. When studying the flow of fluids in different geometries, two-period cylindrical arrays are usually used. For example, two-dimensional flow in two-period cylindrical arrays was studied by Alcocer and Singh et al. They demonstrated a non-monotonic dependence of permeability on aspect ratio [160,161].

In almost all porous media, the main driving force of the fluid is the capillarity. The lateral flow of a fluid in a microfluidic channel usually follows the Washburn equation:

(10) $$l=\sqrt{\frac{\gamma \mathrm{COS}\theta }{2\eta }}\mathrm{rt}$$

where γ is the surface tension, θ is the contact angle, r is the average pore radius, t is the time, η is viscosity of the fluid, and l is the distance. The equation shows that when the temperature and channel width are equal, the distance traveled by the fluid is proportional to the square root of time [155].

### 3.4. Other Relative Models

Based on the previous fluid dynamics, the researchers developed further physical models in different application scenarios to make more accurate physical simulations and calculations of the flow properties of liquids in porous structures.

Chang et al. proposed that the limited accuracy of the Washburn equation is mainly due to the internal cavities of the cellulose fibers that compose the paper [162]. They conducted a combined experimental and theoretical study, where experimental measurements of the internal structure of cellulose fibers were carried out and a mathematical model of liquid absorption was developed by considering the flow through the inner pores of the fibers.

In this model, the relationship between the length of the liquid absorbed by the cylindrical tube *ll*) and the time of inflow into the slit (*t*) is:

(11) $$l^{*}\left(t^{*}\right)l^{*\prime }\left(t^{*}\right)+\frac{1}{2}\psi f\left(t^{*}\right)=\frac{1}{2}$$

where

(12) $$l^{*}=l/l_{c}$$

(13) $$t^{*}=t/t_{c}$$

(14) $$f\left(t^{*}\right)=\left\{\begin{array}{c} \int_{0}^{t^{*}}\left(\int_{\tau^{*}}^{t^{*}}\frac{l^{*\prime }\left(\tau^{*}\right)}{\sqrt{t^{*}-\tau^{*}}}d\tau^{*}\right)l^{*\prime }\left(\tau^{*}\right)d\tau^{*},t^{*}<1\\ \int_{0}^{t^{*}-1}\left(\int_{\tau^{*}-1}^{t^{*}}\frac{l^{*\prime }\left(\tau^{*}\right)}{\sqrt{t^{*}-\tau^{*}}}d\tau^{*}\right)l^{*\prime }\left(\tau^{*}\right)d\tau^{*}+\int_{t^{*}-1}^{t^{*}}\left(\int_{\tau^{*}}^{t^{*}}\frac{l^{*\prime }\left(\tau^{*}\right)}{\sqrt{t^{*}-\tau^{*}}}d\tau^{*}\right)l^{*\prime }\left(\tau^{*}\right)d\tau^{*},\;t^{*}>1 \end{array}\right.$$

(15) ψ=eH/πR

*Ψ* is the volume ratio of slit to the circular tube, which corresponds to the volume ratio of intra-fiber pores to inter-fiber pores. This reveals the key physical reason for the limited accuracy of the Washburn equation for paper capillary flow. The model agrees well with the experimental results and provides a new explanation for the flow of porous media with size pores.

Cao et al. proposed a mechanism to describe the movement of erythrocytes on paper [163]. The morphology and formation of blood stains on paper substrates and the factors influencing the typical shape of blood stains were investigated at the macroscopic and cellular levels, reporting that the formation of ring-shaped red blood cell stains on paper is mainly the result of a combination of capillary core suction, filtration and evaporation fluxes, and it is influenced by the fiber structure, *RBC* incubation time, relative humidity, and paper additives. The dynamic transport behavior of erythrocytes in paper was quantified by the equation:

(16) $$\rho =\frac{VC_{RBC}}{V_{c}}/\left(\pi \frac{d^{2}}{4}\right)$$

where ρ is the density of red blood cells, d is the wetting diameter, *V* is the volume of *RBC* samples deposited on the paper, *Vc* is the average red blood cell volume of individual red blood cells, and *C_RBC_* is the red blood cell volume concentration.

## 4. Microfluidic Devices Based on Porous Media for Biomedical Analysis

With the aging of the population and the improvement of people’s quality of life, the demand for health testing and medical services has grown rapidly in recent years. In order to effectively solve this problem, the prevention, diagnosis, treatment and management of various diseases have become effective solutions. Biosystems analysis and biomolecular assays in biomedical analysis have become the most important part of healthcare testing management. Traditional medical diagnosis requires expensive specialized testing equipment and complex operations, which can be difficult to access for resource-limited areas. Microfluidic devices, due to their small size, portability, and rapid diagnostic capabilities, offer the potential to significantly reduce costs and expand the scope of medical diagnosis. For example, microfluidic based point-of-care (POC) equipment is used for rapid analysis and testing outside the laboratory [164,165,166]. Wearable and miniaturized real-time detection devices have become a trend in the development of healthcare devices. Microfluidic devices based on porous media offer great potential for next-generation health monitoring and medical services because of their high specific surface area, portability, and affordability.

### 4.1. Porous Media-Based Microfluidic Devices for Biomedical Analysis

Body fluids include tears, sweat, urine, saliva and blood, which are ideal sources for medical diagnostic sampling because of the large number of small biological molecules they contain [7,167]. For example, saliva contains proteins, DNA and many microorganisms, sweat contains many metabolites such as sodium ions, calcium ions, lactic acid and urea, and glucose is present in many body fluids such as blood, sweat and tissue fluids [8].

**Figure 5:**
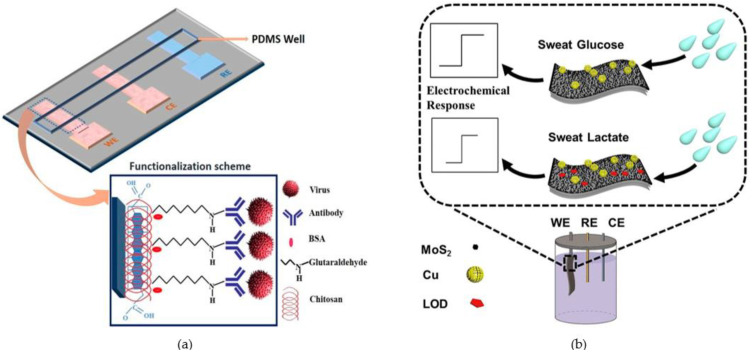
(**a**) The paper-based immunosensor with a PDMS well; (**b**) Graphene paper-based platform for sweat glucose and lactate sensing. (**a**) Reproduced with permission. Ref. [181] Copyright 2017, Sensors (Basel). (**b**) Reproduced with permission. Ref. [189] Copyright 2018, Anal Biochem.

Saliva, as the most readily available body fluid that does not require in vivo extraction, has become the hottest research area in non-invasive microfluidic devices for diagnosis using body fluid samples [168,169,170]. Saliva is a complex mixture containing various proteins from blood (C-reactive protein, α-1B glycoprotein, etc.) and glucose from sweat, among other components. Therefore, through different biomarkers in saliva, it is possible to reflect the body’s systemic health status (cardiovascular disease, cancer, diabetes, etc.) [171]. Glucose levels in saliva can replace blood as a routine screening tool for diabetes because of its correlation with blood glucose [171,172,173,174]. Therefore, in recent years, microfluidic devices based on glucose detection in porous media have become a research hotspot [175,176,177,178]. Jia et al. first proposed a point-of-care glucose sensor with fiber paper integrated with graphene oxide [179]. The porous structure in the medium effectively increased the absorption rate of the reagent, thus improving the reaction efficiency and the uniformity of the color distribution of glucose. Nitrite is a toxic class 2A carcinogen commonly found in spoiled and expired foods, food additives and preservatives. Therefore, the detection and monitoring of nitrite in humans is essential for the protection of human health. Zhang et al. developed colorimetric microfluidic paper-based analysis devices based on electrokinetic stacking that can be used to detect nitrite concentrations in saliva [180]. Saliva was passed on paper-based microchannels coated with Griess reagent, and the microfluidic paper-based analysis devices was shown to have a linear response range of 0.075–1.0 μg/mL with a limit of detection of 73 ng/mL. Devarakonda et al. developed a microfluidic point-of-care device for detecting influenza virus in saliva samples (Figure 5a). The device was coated with superhydrophobic silica nanoparticles on modified porous fiber paper, and then, the electrodes were patterned by a printing technique using single-walled carbon nanotubes and chitosan modifications. This microfluidic device was shown to have good detectability of H1N1 virus [181]. Izabela et al. probed the lithium ion concentration in saliva by a microfluidic wire-based device. The combination of extractant and porous media was used to effectively improve the sensitivity of lithium ion extraction and device [182].

For non-invasive devices, sweat is another important body fluid to detect because of its biomarkers such as electrolytes, glucose, protein, and lactate [34]. Since sweat is only produced when the body is exercising or when the room temperature is high, the breathability and comfort of microfluidic devices become the direction to focus on sweat [183,184,185,186]. Microfluidic sensors with porous media have good breathability and can collect sweat quickly and efficiently through capillaries. The ion concentration in sweat can reflect the balance of water and various substances in the human body [187]. Maryam et al. presented a microfluidic paper-based analysis device to measure chloride ion content in sweat. A scale in the microchannel was used to quantify adsorbed chloride ions and thus detect cystic fibrosis [188]. Creatine levels in sweat can reflect the recovery of the body after exercise. The POC device is capable of analyzing a small sample (2 μL) within one minute without requiring any electronical-based readout. This makes it a suitable option for providing CF diagnosis in developing countries and resource-limited areas. Curto et al. proposed the preparation of a flexible microfluidic platform based on ionic liquid polymer gel. This portable, wearable microfluidic device can provide real-time feedback on sweat composition during human exercise [31]. Wang et al. reported a highly flexible graphene-based paper platform for the detection of lactate in sweat, consisting of Cu submicron buds deposited on free-standing graphite paper and monolayer molybdenum disulfide crystals [180] (Figure 5b). The sensor displays a linear lactate detection range of 0.01–18.4 mM. 

Torul et al. mapped microfluidic channels on the surface of porous cellulose membranes by the wax-printing technique for the preparation of microfluidic devices for the detection of glucose in blood. When blood flows in, a large number of blood cells, etc., will be retained on the nitrocellulose membrane, and small molecules such as glucose enter the detection zone smoothly and are characterized by surface-enhanced Raman scattering spectroscopy [190]. Table 2 briefly reviews several other microfluidic devices proposed in the literature for the detection of porous media in body fluid samples (Table 2).

### 4.2. Microfluidic Devices for Other Biomedical Analysis Applications

**Figure 6:**
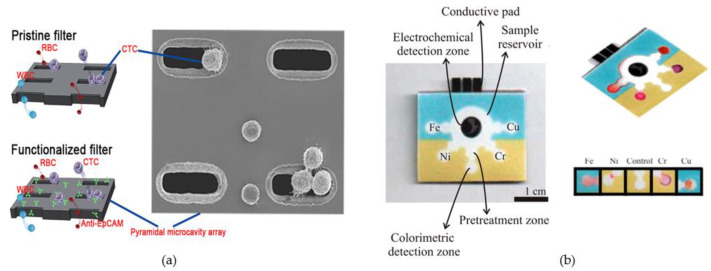
(**a**) Filter process of the pristine filter and the functionalized filter; (**b**) microfluidic paper-based analytical device for toxic metals detection. (**a**) Reproduced with permission. Ref. [225] Copyright 2019, Anal Chim Acta. (**b**) Reproduced with permission. Ref. [40] Copyright 2014, Anal Chem.

Microfluidic devices have important applications in other biomedical aspects in addition to the analysis and detection of body fluid components [7,216,217]. The transport and delivery of specific fluids (drugs, cells, etc.) using microfluidic channels with porous media has become a hot research direction in recent years due to the unique physical and chemical properties of microfluidic channels [218,219,220,221]. Nanoparticles can encapsulate various drugs to improve their stability and solubility, transport and release them through microfluidic channels to make the transport process controllable and reduce their toxicity [222]. For example, Chen et al. developed a PDMS microfluidic chip for generating polymer nanoparticles loaded with curcumin by generating a gas segmented liquid plug. The controllable nanoparticle diameter can be achieved by regulating the flow rate of liquid in the channel [223]. Microfluidic devices also have the ability to capture, align and manipulate cells. Cell separation and capture can be achieved by preparing arrays of porous microstructures, such as separating cancer cells from healthy cells for cancer diagnosis [224,225,226,227]. Yin et al. prepared the first pyramidal porous array to capture specific cells [225] (Figure 6a). In this platform, micro-cavity filter arrays were designed to capture and enrich circulating tumor cells from primary blood samples and ensure the escape of red blood cells and leukocytes. It was demonstrated that less than 0.003% of leukocytes remained in the pyramidal microarrays while ensuring the capture of more than 83% of circulating tumor cells. Microfluidic devices based on porous structures have unique advantages in chemical and biomolecular screening due to their inherent structural properties. Yao et al. used PDMS sub-nanoliter pore arrays to isolate and analyze circulating tumor cells in whole blood, and only a few circulating tumor cells possessed the phenotype of metastatic potential and secreted protein hydrolases [226]. The screening and detection of cells by microfluidics based on porous structures can improve our understanding and analysis of cells; thus, disease screening and development provide more new options. Heavy metals are a group of substances that seriously pollute the environment and cause harm to the human body. For example, lead ions can cause great damage to the kidneys and lead to abnormal neurodevelopment in humans, and cadmium ions can affect the proliferation and differentiation of human cells [228]. Therefore, the detection of heavy metal ions has become an important research direction to protect human health. Rattanarat et al. developed an inexpensive and mass-produced paper-based microfluidic assay device that can detect six different metals by the separation of colorimetric and electrochemical methods [40] (Figure 6b). In the colorimetric detection mode, the detection limits of the platform were 0.12, 0.75, 0.75 and 0.75 μg for Cr, Fe, Cu and Ni, and 0.25 ng (1 μg/L) was obtained for Pb and Cd when analyzed on 2 mm and 10 mm filter punches, respectively. These low-cost portable detection devices can easily and quickly detect toxic metals. Porous materials possess unique properties for fluid flow and separation, and they can also serve as scaffolds to mimic the process of cell proliferation and differentiation in biological tissues for 3D cell culture. This makes them highly versatile for use in various applications, including cell physiology, tumor models, and drug delivery [229,230,231]. Yu et al. developed a PMMA master mold with 3D undulated microtopographies, which was then used to create a PDMS production mold [232]. Gelatin chondroitin sulfate-6 sulfate (Gel-C6S-HA) was then filled into the PDMS mold and freeze-dried to obtain a porous scaffold. Newborn human fibroblasts (NHF) were cultured on the scaffold surface for up to 7 days, demonstrating the biocompatibility of the scaffold and showing unique cell responses at a macroscopic level with biomimetic morphology. Li et al. prepared a method for constructing aligned porous scaffolds for 3D cell culture that does not require cross-linking agents [233]. The porosity of the porous foam was adjusted by controlling the amount of NaCl and the ratio of the oil phase. The foam scaffold does not possess cytotoxicity. Mouse fibroblast cells NIH/3T3 were grown in the surface and internal structures of the foam, which proved its promising application in bioadaptive 3D scaffolds for tissue engineering. Extracellular vesicles derived from tumor cells, which can be stably detected in various body fluids and reflect the tumor burden status in real time, are considered a promising tool for liquid biopsy. Li et al. functionalized a 3D porous PDMS sponge structure with CD9 antibody to capture extracellular vesicles imported into a microfluidic chip [234]. This work is based on a 3D porous microfluidic chip platform and is used as a new non-invasive diagnostic tool for the early detection of colorectal cancer.

## 5. Conclusions and Perspective

This paper reviews the recent advances in microfluidic devices based on porous discontinuous media, covering from the preparation and principles of porous media to the application of the devices in biomedical fields. Microfluidics based on discontinuous media is currently of great value in the biomedical field due to comprehensive and precise theoretical studies and technological innovations. Despite the many exciting research and innovations, many challenges remain.

The first problem should focus on the materials and preparation methods of discontinuous porous media. The existing preparation methods generally focus on technical methods such as photolithography, etching and printing. However, these methods all have relatively more steps and longer preparation cycles, which are not conducive to reducing the cost of the device and the mass production of the device. In addition, most of the current material choices for porous media are focused on PDMS, paper and some etched metals, which cannot have both flexibility and stability in some special environments. Therefore, more new materials with excellent performance need to be explored and applied.

The second issue focuses on the fundamental study of microfluidics. Due to the relatively complex composition of fluids in practical applications, many fluid mechanics with conditional limitations and fundamentals are no longer applicable. The study of surface tension, rheological behavior, interfacial stability and hysteresis effects of impure components needs to be further explored. In addition, the theory of more complex interfacial parameters with time and space needs to be explored more deeply, considering the possible adsorption of certain interfacially active components.

The last issue concerns the practical applications in the medical and healthcare field. Currently, most microfluidic devices based on porous structures are prepared in research institutions or in university laboratories. This firstly limits the access to microfluidics, and the separation between laboratory and commerce prevents most of the research from realizing its market applications and commercial value. Secondly, the laboratory has sophisticated equipment and stable testing environment, which makes many excellent performance and stable data uncontrollable in practical applications. Therefore, there is a need to reconcile research results with commercial manufacturing.

Despite some of the current issues that need to be addressed, microfluidics has found an exciting and widespread use in biomedical analysis. The emergence of advanced technologies such as artificial intelligence, deep learning, and simulation techniques provides tremendous opportunities for the development of microfluidics based on porous media. Artificial intelligence can process and analyze data extremely quickly to assist clinicians in making accurate and timely diagnoses. Deep learning and big data can effectively analyze global user-generated data and make trend predictions. Multi-disciplinary collaboration is also a welcome trend. Numerous research institutions are already producing exciting results through the assistance and deep collaboration of researchers from different fields, which bridges the gap between material synthesis, theoretical research and applications. We firmly believe that the future will bring more exciting achievements in microfluidics based on porous materials.

## Figures and Tables

**Table 1:** Comparison of preparation methods and their advantages and disadvantages for different materials.

Material	Advantages	Limitations	Fabrication Technique	Advantages	Limitations	Refs.
PDMS	Easy fabrication, high flexibility, and thermal stability	Poor long-term stability	Soft lithography	High-resolution, facile fabrication	Difficulty in fabrication over large areas	[112,113]
			Sacrificial template	Low-cost, size adjustable	Difficulty in removing templates, uneven pore distribution may exist	[35,36,114]
			Track etching	Allows the formation of uniform pore sizes and controlled pore densities	Time consuming	[115]
			Gas foaming technique	Facile and eco-friendly fabrication procedures	Poor control of pore size and porosity	[116]
			Laser micromachining	Simple, fast and low cast, high precision	The principle of interaction between the material and the laser is not entirely clear	[117,118]
			3D printing	Desirable pore size and porosity	Relatively high cost, low fabrication efficiency	[36,119]
PMMA	Low processing cost, good mechanical properties,	Poor biocompatibility	Hot embossing	Facile, low cast	Requires high temperature and pressure conditions	[86,120]
			Direct laser writing	Short cycle time of production	Limited resolution	[121,122]
			Injection molding	Fast, high production efficiency	High cost of mold equipment	[123]
Paper	Cost-effective, simple, disposable, and portable	Channel size is not easy to control and not standardized, Auto-fluorescence interference	Photolithography	High resolution	Difficulty in fabrication process, time consuming, high cost	[1,124]
			Printing	Low cast, simple operation procedures	Low resolution	[100,125]
			Etching	Low cast	Low resolution and complexity, difficult to fabricate high-density microchannel networks	[126,127]
			Embossing	Complex microfluidic networks can be prepared	The preparation process is complicated	[100,128]
Hydrogels	Biocompatibility, mechanical tenability	Complex preparation process	Templated-assisted	Control of the porous properties, morphology, and structure	Time consuming, complex process of template leaching	[129]
			Freeze drying	Suitable for almost any material	High energy consumption, inability to precisely control porosity	[130,131]
			3D printing	Rapid and can produce complex, three-dimensional structures.	Limited resolution	[132,133]
Textile	Excellent biocompatibility	Non-standardized, not easy to mass produce	Spinning	Facile fabrication	Difficult to precise modeling	[134,135]
			Electrospinning	Can prepare nano-scale microfiber	Inability to precisely control fiber diameter	[20]

**Table 2:** Summarizes the porous based microfluidics for detection on body fluid samples.

Ref. and Years	Materials	Fabrication Methods	Detection Methods	Target and Sample Matrices	Detection Limit
[170] Patarajarin et al., 2022	Paper	Wax printing	Antigen test	SARS-CoV-2 (Saliva)	1 fg/μL
[184] Hong et al., 2022	Hybrid Janus Membrane	Roller-assisted liquid printing.	Electrochemical	Glucose and lactate (sweat).	0.15 μL
[185] Li et al., 2022	Hydrogel paper	Self-assembled	Electrochemical	Glucose (sweat)	10.3 μM
[186] Mogera et al., 2022	Paper	Cutting	Surface-enhanced Raman spectroscopy (SERS)	Uric acid (sweat)	1 μM
[191] Li et al., 2021	Paper	Printing	Electrochemical	Glucose and lactate (sweat)	17.05 μM
[192] Bagheri et al., 2021	Paper	Wax printing	Electrochemical	Copper ions (sweat and serum)	3 ppb
[193] Fiore et al., 2023	Paper	Waxing printing	Electrochemical	Cortisol (sweat)	101 mM
[194] Weng et al., 2022	Paper	Screen-printing	Electrochemical	Cortisol (sweat)	0.1 nM
[195] Singh et al., 2022	Paper	Cutting	Electrochemical	Glucose (sweat)	0.5 μM
[196] Fabiani et al., 2022	Paper	Wax printing	Electrochemical	SARS-CoV-2 (saliva)	0.1 ug/mL
[197] Moon et al., 2022	PVA-based hydrogel	Sacrificial template	Electrochemical	βHydroxybutyrate (sweat)	62 μM
[198] Gunatilake et al., 2021	Nanotubes alginate hydrogel	Freeze-drying	Colorimetric	Glucose (sweat)	0.8 mM
[199] Guzman et al., 2020	Hydrogel	Sacrificial template	Colorimetric	Lipocalin-1 (tear)	1 ng/mL
[200] Xu et al., 2021	PEDOT:PSS hydrogel	Sacrificial template	Electrochemical	Uric acid (sweat)	1.2 μM
[201] Siripongpreda et al., 2021	Hydrogel	Matrix deposition	Colorimetric	Glucose (sweat)	25 μM
[202] Yeung et al., 2022	Graphene	Chemical vapor deposition	Electrochemical	Na+ (sweat)	10 mM
[203] Yoon et al., 2020	Graphene	Laser-induced	Electrochemical	Glucose (sweat)	300 nM
[204] Wang et al., 2021	Hydrogels	Cross-linking	Strain sensor	NaCl (sweat)	0.15 μL
[205] Saha et al., 2021	Paper	Cutting	Colorimetry	Lactate (sweat)	20 mM
[47] Baretta et al., 2023	Hydrogel	Template	Electrochemical	Glucose (serum)	1 mM
[206] Liu et al., 2021	PDMS	Template	Electrochemical	Cortisol (sweat)	0.3 fg/mL
[207] Li et al., 2023	Graphene	Hydrothermal	Electrochemical	Glucose (sweat)	2.45 μM
[208] Xuan et al., 2018	Graphene	Laser-induced	Electrochemical	Glucose (sweat)	5 μM
[209] Kil et al., 2022	Graphene inks	Printing	Electrochemical	Na+ (sweat)	9.1 × 10^−7^ M
[210] Liu et al., 2021	PEN and SFNFs	Hybridization material strategy	Electrochemical	Glucose (sweat)	2 mM
[211] Xu et al., 2021	Reduced graphene oxide	Electrostatic self-assembly	Electrochemical	Glucose (sweat)	3.7 μM
[212] Poletti et al., 2021	Graphene oxide	Chemical functionalization	Electrochemical	Glucose and lactate (sweat)	32/68 nM
[173] Chakraborty et al., 2020	CuO	Hydrothermal synthesis	Electrochemical	Enzyme-less glucose (saliva)	0.41 μM
[213] Park et al., 2022	Platinum nanozyme-hydrogel composite	Photopolymerization	Colorimetry	Glucose (serum)	3.9 μM
[214] Elancheziyan et al., 2023	Co-PM-NDGPC/SPE	Single-step electrodeposition	Electrochemical	Glucose (blood)	7.9 μM
[215] Yao et al., 2022	ZGC PLNPs	Self-assembly	Fluorescence analysis	Dopamine (serum)	0.001 μM

## Data Availability

Data sharing not applicable No new data were created or analyzed in this study.

## References

[1] Ma J., Yan S., Miao C., Li L., Shi W., Liu X., Luo Y., Liu T., Lin B., Wu W. (2019). Paper Microfluidics for Cell Analysis. Adv. Healthc. Mater..

[2] Zhang L., Tan Q., Fan J., Sun C., Luo Y., Liang R., Qiu J. (2023). Microfluidics for chiral separation of biomolecules. TrAC Trends Anal. Chem..

[3] Dungchai W., Chailapakul O., Henry C.S. (2009). Electrochemical Detection for Paper-Based Microfluidics. Anal. Chem..

[4] Ayuso J.M., VirumbralesMuñoz M., Lang J.M., Beebe D.J. (2022). A role for microfluidic systems in precision medicine. Nat. Commun..

[5] Terry S.C., Jerman J.H., Angell J.B. (1979). A gas chromatographic air analyzer fabricated on a silicon wafer. IEEE Trans. Electron Dev..

[6] Yang S.M., Lv S., Zhang W., Cui Y. (2022). Microfluidic Point-of-Care (POC) Devices in Early Diagnosis: A Review of Opportunities and Challenges. Sensors.

[7] Yang Y., Chen Y., Tang H., Zong N., Jiang X. (2020). Microfluidics for Biomedical Analysis. Small Methods.

[8] Tseng C., Kung C., Chen R., Tsai M., Chao H., Wang Y., Fu L. (2021). Recent advances in microfluidic paper-based assay devices for diagnosis of human diseases using saliva, tears and sweat samples. Sens. Actuators B Chem..

[9] Yang Z., Zhou Z., Si T., Zhou Z., Zhou L., Chin Y.R., Zhang L., Guan X., Yang M. (2023). High Throughput Confined Migration Microfluidic Device for Drug Screening. Small.

[10] Li S., Ma Z., Cao Z., Pan L., Shi Y. (2020). Advanced Wearable Microfluidic Sensors for Healthcare Monitoring. Small.

[11] Mashaghi S., Abbaspourrad A., Weitz D.A., van Oijen A.M. (2016). Droplet microfluidics: A tool for biology, chemistry and nanotechnology. TrAC Trends Anal. Chem..

[12] Zheng L., Cai G., Wang S., Liao M., Li Y., Lin J. (2019). A microfluidic colorimetric biosensor for rapid detection of Escherichia coli O157:H7 using gold nanoparticle aggregation and smart phone imaging. Biosens. Bioelectron..

[13] Park J., Park J. (2019). Finger-Actuated Microfluidic Display for Smart Blood Typing. Anal. Chem..

[14] Zhang T., Ratajczak A.M., Chen H., Terrell J.A., Chen C. (2022). A Step Forward for Smart Clothes─Fabric-Based Microfluidic Sensors for Wearable Health Monitoring. ACS Sens..

[15] Lim H., Lee S.M., Park S., Choi C., Kim H., Kim J., Mahmood M., Lee Y., Kim J., Yeo W. (2022). Smart bioelectronic pacifier for real-time continuous monitoring of salivary electrolytes. Biosens. Bioelectron..

[16] Zhang W., Hou C., Li Y., Zhang Q., Wang H. (2020). Microfluidic spinning of editable polychromatic fibers. J. Colloid Interface Sci..

[17] Yu Y., Shang L., Guo J., Wang J., Zhao Y. (2018). Design of capillary microfluidics for spinning cell-laden microfibers. Nat. Protoc..

[18] Cheng R., Liang Z.B., Zhu L., Li H., Zhang Y., Wang C.F., Chen S. (2022). Fibrous Nanoreactors from Microfluidic Blow Spinning for Mass Production of Highly Stable Ligand-Free Perovskite Quantum Dots. Angew. Chem. Int. Ed..

[19] Guo J., Zhang H., Zhang H., Chen H., Gu Z., Zhang D., Zhao Y. (2022). Jellyfish Tentacle-Inspired Hydrogel Microfibers Implanted with Discrete Structural Color Microsphere Tactile Sensing Units. Adv. Fiber Mater..

[20] Cheng J., Jun Y., Qin J., Lee S. (2017). Electrospinning versus microfluidic spinning of functional fibers for biomedical applications. Biomaterials.

[21] Chen T., Huang C., Wang Y., Wu J. (2022). Microfluidic methods for cell separation and subsequent analysis. Chin. Chem. Lett..

[22] Salafi T., Zeming K.K., Lim J.W., Raman R., Seah A.W.R., Tan M.P., Zhang Y. (2019). Portable Smartphone-Based Platform for Real-Time Particle Detection in Microfluidics. Adv. Mater. Technol..

[23] Deng Y., Davis S.P., Yang F., Paulsen K.S., Kumar M., Sinnott Devaux R., Wang X., Conklin D.S., Oberai A., Herschkowitz J.I. (2017). Inertial Microfluidic Cell Stretcher (iMCS): Fully Automated, High-Throughput, and Near Real-Time Cell Mechanotyping. Small.

[24] Chen Y., Schoeler U., Huang C.B., Vollmer F. (2018). Combining Whispering-Gallery Mode Optical Biosensors with Microfluidics for Real-Time Detection of Protein Secretion from Living Cells in Complex Media. Small.

[25] Zhao W., van den Berg A. (2008). Lab on paper. Lab A Chip.

[26] Holman J.B., Shi Z., Fadahunsi A.A., Li C., Ding W. (2023). Advances on microfluidic paper-based electroanalytical devices. Biotechnol. Adv..

[27] Banik S., Uchil A., Kalsang T., Chakrabarty S., Ali M.A., Srisungsitthisunti P., Mahato K.K., Surdo S., Mazumder N. (2022). The revolution of PDMS microfluidics in cellular biology. Crit. Rev. Biotechnol..

[28] Shangguan J., Liu Y., Pan J., Xu B., Xu J., Chen H. (2017). Microfluidic PDMS on paper (POP) devices. Lab A Chip.

[29] Comina G., Suska A., Filippini D. (2014). PDMS lab-on-a-chip fabrication using 3D printed templates. Lab A Chip.

[30] Chen C., Meng H., Guo T., Deshpande S., Chen H. (2022). Development of Paper Microfluidics with 3D-Printed PDMS Barriers for Flow Control. ACS Appl. Mater. Interfaces.

[31] Curto V.F., Fay C., Coyle S., Byrne R., O’Toole C., Barry C., Hughes S., Moyna N., Diamond D., BenitoLopez F. (2012). Real-time sweat pH monitoring based on a wearable chemical barcode micro-fluidic platform incorporating ionic liquids. Sens. Actuators B Chem..

[32] Wu G., Tan P., Wu X., Peng L., Cheng H., Wang C.F., Chen W., Yu Z., Chen S. (2017). High-Performance Wearable Micro-Supercapacitors Based on Microfluidic-Directed Nitrogen-Doped Graphene Fiber Electrodes. Adv. Funct. Mater..

[33] Huang Y., Zhong M., Shi F., Liu X., Tang Z., Wang Y., Huang Y., Hou H., Xie X., Zhi C. (2017). An Intrinsically Stretchable and Compressible Supercapacitor Containing a Polyacrylamide Hydrogel Electrolyte. Angew. Chem. Int. Ed..

[34] Nyein H.Y.Y., Bariya M., Kivimäki L., Uusitalo S., Liaw T.S., Jansson E., Ahn C.H., Hangasky J.A., Zhao J., Lin Y. (2019). Regional and correlative sweat analysis using high-throughput microfluidic sensing patches toward decoding sweat. Sci. Adv..

[35] Choi S.J., Kwon T.H., Im H., Moon D.I., Baek D.J., Seol M.L., Duarte J.P., Choi Y.K. (2011). A polydimethylsiloxane (PDMS) sponge for the selective absorption of oil from water. ACS Appl. Mater. Interfaces.

[36] Montazerian H., Mohamed M.G.A., Montazeri M.M., Kheiri S., Milani A.S., Kim K., Hoorfar M. (2019). Permeability and mechanical properties of gradient porous PDMS scaffolds fabricated by 3D-printed sacrificial templates designed with minimal surfaces. Acta Biomater.

[37] Hamedi M.M., Ainla A., Guder F., Christodouleas D.C., FernandezAbedul M.T., Whitesides G.M. (2016). Integrating Electronics and Microfluidics on Paper. Adv. Mater..

[38] Lee Y.Y., Kang H.Y., Gwon S.H., Choi G.M., Lim S.M., Sun J.Y., Joo Y.C. (2016). A Strain-Insensitive Stretchable Electronic Conductor: PEDOT:PSS/Acrylamide Organogels. Adv. Mater..

[39] Pendergraph S.A., Bartlett M.D., Carter K.R., Crosby A.J. (2012). Opportunities with fabric composites as unique flexible substrates. ACS Appl. Mater. Interfaces.

[40] Rattanarat P., Dungchai W., Cate D., Volckens J., Chailapakul O., Henry C.S. (2014). Multilayer paper-based device for colorimetric and electrochemical quantification of metals. Anal. Chem..

[41] Whitesides G.M. (2006). The origins and the future of microfluidics. Nature.

[42] Khan S.M., Gumus A., Nassar J.M., Hussain M.M. (2018). CMOS Enabled Microfluidic Systems for Healthcare Based Applications. Adv. Mater..

[43] Wang H., Wang X., Cheng J. (2022). Bionic Enzyme-Assisted Ion-Selective Amperometric Biosensor Based on 3D Porous Conductive Matrix for Point-of-Care Nitrite Testing. ACS Nano.

[44] Vaquer A., AdroverJaume C., Clemente A., Iglesias A., López M., Martínez R., Roig I.M., Cosío B.G., de la Rica R. (2023). Immunosensors made of polymer-infused porous paper for the non-invasive detection of airways cytokines trapped by porous face masks. Sens. Actuators B Chem..

[45] Xu D., Shen Z., Wang G., Wei L., Gao X., Dong H., Wang G., Sun X., Li F., Guo Y. (2023). Dual-catalytic colorimetric biosensor based on double-active Fe@Co-N stellate porous carbon and DNAzyme for simultaneous detection of tetracycline antibiotics. Sens. Actuators B Chem..

[46] Zhang Y., Jiang Y., Duan Z., Wu Y., Zhao Q., Liu B., Huang Q., Yuan Z., Li X., Tai H. (2022). Edge-enriched MoS2 nanosheets modified porous nanosheet-assembled hierarchical In2O3 microflowers for room temperature detection of NO2 with ultrahigh sensitivity and selectivity. J. Hazard. Mater..

[47] Baretta R., Raucci A., Cinti S., Frasconi M. (2023). Porous hydrogel scaffolds integrating Prussian Blue nanoparticles: A versatile strategy for electrochemical (bio)sensing. Sens. Actuators B Chem..

[48] Li J., Gao J., Guo T., Huang X., Zhang X., Xu C., Xue H. (2019). Hierarchically Porous Copolymer Film as Immobilization Matrix for Phenol Biosensor with High Sensitivity. ACS Appl. Polym. Mater..

[49] Zhou X., Li P., Wu X., Lin X., Zhao L., Huang H., Wu J., Cai H., Xu M., Zhou H. (2022). Multifunctional biosensor constructed by Ag-coating magnetic-assisted unique urchin core porous shell structure for dual SERS enhancement, enrichment, and quantitative detection of multi-components inflammatory markers. Biosens. Bioelectron..

[50] Mu X., Bertron T., Dunn C., Qiao H., Wu J., Zhao Z., Saldana C., Qi H. (2017). Porous polymeric materials by 3D printing of photocurable resin. Mater. Horiz..

[51] Chen X., Cui D., Zhang L. (2009). Isolation of plasma from whole blood using a microfludic chip in a continuous cross-flow. Chin. Sci. Bull..

[52] Yuan Y., Yang Y., Faheem M., Zou X., Ma X., Wang Z., Meng Q., Wang L., Zhao S., Zhu G. (2018). Molecularly Imprinted Porous Aromatic Frameworks Serving as Porous Artificial Enzymes. Adv. Mater..

[53] Balakrishnan H.K., Dumée L.F., Merenda A., Aubry C., Yuan D., Doeven E.H., Guijt R.M. (2023). 3D Printing Functionally Graded Porous Materials for Simultaneous Fabrication of Dense and Porous Structures in Membrane-Integrated Fluidic Devices. Small Struct..

[54] Tu T., Liang B., Zhang S., Li T., Zhang B., Xu S., Mao X., Cai Y., Fang L., Ye X. (2021). Controllable Patterning of Porous MXene (Ti_3_C_2_) by Metal-Assisted Electro-Gelation Method. Adv. Funct. Mater..

[55] Hua E., Zhang Y., Yun K., Pan W., Liu Y., Li S., Wang Y., Tu R., Wang M. (2022). Whole-Cell Biosensor and Producer Co-cultivation-Based Microfludic Platform for Screening Saccharopolyspora erythraea with Hyper Erythromycin Production. ACS Synth. Biol..

[56] Liu L., Liu Z., Wang Q., Wang F., Li J., Xie R., Ju X., Wang W., Pan D., Chu L. (2023). Porous functional materials with excellent solar-thermal and electro-thermal properties for desalination of saline water. Sep. Purif. Technol..

[57] Liu X., Li Y., Chen Z., Yang H., Cai Y., Wang S., Chen J., Hu B., Huang Q., Shen C. (2023). Advanced porous nanomaterials as superior adsorbents for environmental pollutants removal from aqueous solutions. Crit. Rev. Environ. Sci. Technol..

[58] Zhu Y., Zhang X., Sun L., Wang Y., Zhao Y. (2023). Engineering Human Brain Assembloids by Microfluidics. Adv. Mater..

[59] Hou C., Gu Y., Yuan W., Zhang W., Xiu X., Lin J., Gao Y., Liu P., Chen X., Song L. (2023). Application of microfluidic chips in the simulation of the urinary system microenvironment. Mater. Today Bio.

[60] Shang L., Cheng Y., Zhao Y. (2017). Emerging Droplet Microfluidics. Chem. Rev..

[61] Mery E., Ricoul F., Sarrut N., Constantin O., Delapierre G., Garin J., Vinet F. (2008). A silicon microfluidic chip integrating an ordered micropillar array separation column and a nano-electrospray emitter for LC/MS analysis of peptides. Sens. Actuators B Chem..

[62] Singh N.K., Chung S., Chang A.-Y., Wang J., Hall D.A. (2023). A non-invasive wearable stress patch for real-time cortisol monitoring using a pseudoknot-assisted aptamer. Biosens. Bioelectron..

[63] Chen G., Zheng J., Liu L., Xu L. (2019). Application of Microfluidics in Wearable Devices. Small Methods.

[64] Kashaninejad N., Nguyen N. (2023). Microfluidic solutions for biofluids handling in on-skin wearable systems. Lab A Chip.

[65] Zhou H., Wang H., Lin T., Niu H. (2022). A novel Janus fabric with stable amphibious directional oil transport function. Chem. Eng. J..

[66] Li Y., Fischer R., Zboray R., Boillat P., Camenzind M., Toncelli C., Rossi R.M. (2020). Laser-Engraved Textiles for Engineering Capillary Flow and Application in Microfluidics. ACS Appl. Mater. Amp. Interfaces.

[67] Jung K., Corrigan N., Wong E.H.H., Boyer C. (2022). Bioactive Synthetic Polymers. Adv. Mater..

[68] Gao Y., Ota H., Schaler E.W., Chen K., Zhao A., Gao W., Fahad H.M., Leng Y., Zheng A., Xiong F. (2017). Wearable Microfluidic Diaphragm Pressure Sensor for Health and Tactile Touch Monitoring. Adv. Mater..

[69] Jiang B., White A., Ou W., Van Belleghem S., Stewart S., Shamul J.G., Rahaman S.O., Fisher J.P., He X. (2022). Noncovalent reversible binding-enabled facile fabrication of leak-free PDMS microfluidic devices without plasma treatment for convenient cell loading and retrieval. Bioact. Mater..

[70] Habibey R. (2023). Incubator-independent perfusion system integrated with microfluidic device for continuous electrophysiology and microscopy readouts. Biofabrication.

[71] Wu J., Xu F., Li S., Ma P., Zhang X., Liu Q., Fu R., Wu D. (2019). Porous Polymers as Multifunctional Material Platforms toward Task-Specific Applications. Adv. Mater..

[72] Yu C., Yu C., Cui L., Song Z., Zhao X., Ma Y., Jiang L. (2017). Facile Preparation of the Porous PDMS Oil-Absorbent for Oil/Water Separation. Adv. Mater. Interfaces.

[73] Whitesides G.M., Ostuni E., Takayama S., Jiang X., Ingber D.E. (2001). Soft lithography in biology and biochemistry. Annu. Rev. Biomed. Eng..

[74] Han J.H., Kim C.M., Kim T., Jin S., Kim G.M. (2022). Development of In Situ Microfluidic System for Preparation of Controlled Porous Microsphere for Tissue Engineering. Pharmaceutics.

[75] Pan Y., Chen G., Liu J., Li J., Chen X., Zhu H., Liu G., Zhang G., Jin W. (2022). PDMS thin-film composite membrane fabricated by ultraviolet crosslinking acryloyloxy-terminated monomers. J. Membr. Sci..

[76] Chang S.T., Uçar A.B., Swindlehurst G.R., Bradley R.O., Renk F.J., Velev O.D. (2009). Materials of Controlled Shape and Stiffness with Photocurable Microfluidic Endoskeleton. Adv. Mater..

[77] Kong T., Zhou J., Nie F., Zhang C., Shen F., Dai S., Pan H., Gong L., Zhao L. (2022). Sensitive Organic Vapor Sensors Based on Flexible Porous Conductive Composites with Multilevel Pores and Thin, Rough, Hollow-Wall Structure. Polymers.

[78] Zhao X., Li L., Li B., Zhang J., Wang A. (2014). Durable superhydrophobic/superoleophilic PDMS sponges and their applications in selective oil absorption and in plugging oil leakages. J. Mater. Chem. A.

[79] Thiha A., Ibrahim F., Muniandy S., Dinshaw I.J., Teh S.J., Thong K.L., Leo B.F., Madou M. (2018). All-carbon suspended nanowire sensors as a rapid highly-sensitive label-free chemiresistive biosensing platform. Biosens. Bioelectron..

[80] Liu H., Jing Y., Yu X., Pang D., Zhang Z. (2012). Construction of CdSe/ZnS quantum dot microarray in a microfluidic chip. Sci. China Chem..

[81] Van Giesen L., NeaguMaier G.L., Kwon J.Y., Sprecher S.G. (2016). A microfluidics-based method for measuring neuronal activity in Drosophila chemosensory neurons. Nat. Protoc..

[82] Bai S., Serien D., Hu A., Sugioka K. (2018). 3D Microfluidic Surface-Enhanced Raman Spectroscopy (SERS) Chips Fabricated by All-Femtosecond-Laser-Processing for Real-Time Sensing of Toxic Substances. Adv. Funct. Mater..

[83] Wang H., Zhang Y., Wang W., Ding H., Sun H. (2017). On-chip laser processing for the development of multifunctional microfluidic chips. Laser Photon. Rev..

[84] Shin J., Ko J., Jeong S., Won P., Lee Y., Kim J., Hong S., Jeon N.L., Ko S.H. (2021). Monolithic digital patterning of polydimethylsiloxane with successive laser pyrolysis. Nat. Mater..

[85] Karimi S., Mehrdel P., CasalsTerré J., FarréLlados J. (2020). Cost-effective microfabrication of sub-micron-depth channels by femto-laser anti-stiction texturing. Biofabrication.

[86] Bouchard F., Soldera M., Lasagni A.F. (2023). PMMA Optical Diffusers with Hierarchical Surface Structures Imprinted by Hot Embossing of Laser-Textured Stainless Steel. Adv. Opt. Mater..

[87] Volpe A., Krishnan U., Chiriacò M.S., Primiceri E., Ancona A., Ferrara F. (2021). A Smart Procedure for the Femtosecond Laser-Based Fabrication of a Polymeric Lab-on-a-Chip for Capturing Tumor Cell. Engineering.

[88] Say M.G., Brett C.J., Edberg J., Roth S.V., Söderberg L.D., Engquist I., Berggren M. (2022). Scalable Paper Supercapacitors for Printed Wearable Electronics. ACS Appl. Mater. Interfaces.

[89] Liu H., Qing H., Li Z., Han Y.L., Lin M., Yang H., Li A., Lu T.J., Li F., Xu F. (2017). Paper: A promising material for human-friendly functional wearable electronics. Mater. Sci. Eng. R Rep..

[90] Dong L., Xu C., Li Y., Pan Z., Liang G., Zhou E., Kang F., Yang Q. (2016). Breathable and Wearable Energy Storage Based on Highly Flexible Paper Electrodes. Adv. Mater..

[91] Cai S., Zuo C., Zhang J., Liu H., Fang X. (2021). A Paper-Based Wearable Photodetector for Simultaneous UV Intensity and Dosage Measurement. Adv. Funct. Mater..

[92] Huang H., Lin C., Hua Z., Guo J., Lu D., Ni Y., Cao S., Ma X. (2022). Fabrication of ultrathin, flexible, all-in-one paper supercapacitor with high electrochemical performance based on multi-layer forming in paper sheet formation technology. Chem. Eng. J..

[93] Jia C., Jiang F., Hu P., Kuang Y., He S., Li T., Chen C., Murphy A., Yang C., Yao Y. (2018). Anisotropic, Mesoporous Microfluidic Frameworks with Scalable, Aligned Cellulose Nanofibers. ACS Appl. Mater. Interfaces.

[94] Hallan R., Barkas W.W. (1953). Physical Properties of Paper Pulps: ‘Freeness’ of Paper Pulps and the Forces of Capillary Water Retention. Nature.

[95] Nguyen Q.H., Lee D.H., Nguyen P.T., Le P.G., Kim M.I. (2023). Foldable paper microfluidic device based on single iron site-containing hydrogel nanozyme for efficient glucose biosensing. Chem. Eng. J..

[96] Gao B., Li X., Yang Y., Chu J., He B. (2019). Emerging paper microfluidic devices. Analyst.

[97] Fu E., Wentland L. (2022). A survey of 3D printing technology applied to paper microfluidics. Lab A Chip.

[98] Jia Z., Luo Y., Wang D., Dinh Q.N., Lin S., Sharma A., Block E.M., Yang M., Gu T., Pearlstein A.J. (2021). Nondestructive multiplex detection of foodborne pathogens with background microflora and symbiosis using a paper chromogenic array and advanced neural network. Biosens. Bioelectron..

[99] Raj N., Breedveld V., Hess D. (2019). Fabrication of fully enclosed paper microfluidic devices using plasma deposition and etching. Lab A Chip.

[100] Postulka N., Striegel A., Krauße M., Mager D., Spiehl D., Meckel T., Worgull M., Biesalski M. (2019). Combining Wax Printing with Hot Embossing for the Design of Geometrically Well-Defined Microfluidic Papers. ACS Appl. Mater. Interfaces.

[101] Lin D., Li B., Qi J., Ji X., Yang S., Wang W., Chen L. (2020). Low cost fabrication of microfluidic paper-based analytical devices with water-based polyurethane acrylate and their application for bacterial detection. Sens. Actuators B Chem..

[102] Chiang C.-K., Kurniawan A., Kao C., Wang M. (2019). Single step and mask-free 3D wax printing of microfluidic paper-based analytical devices for glucose and nitrite assays. Talanta.

[103] Zhang Y., Yang H., Cui K., Zhang L., Xu J., Liu H., Yu J. (2018). Highly conductive and bendable gold networks attached on intertwined cellulose fibers for output controllable power paper. J. Mater. Chem. A.

[104] Carrilho E., Martinez A.W., Whitesides G.M. (2009). Understanding wax printing: A simple micropatterning process for paper-based microfluidics. Anal. Chem..

[105] Mani V., Kadimisetty K., Malla S., Joshi A.A., Rusling J.F. (2013). Paper-based electrochemiluminescent screening for genotoxic activity in the environment. Env. Sci. Technol..

[106] Guo J., Yu Y., Cai L., Wang Y., Shi K., Shang L., Pan J., Zhao Y. (2021). Microfluidics for flexible electronics. Mater. Today.

[107] Shin S., Hyun J. (2017). Matrix-Assisted Three-Dimensional Printing of Cellulose Nanofibers for Paper Microfluidics. ACS Appl. Mater. Interfaces.

[108] Khan M.J., Zhang J., Guo Q. (2016). Covalent/crystallite cross-linked co-network hydrogels: An efficient and simple strategy for mechanically strong and tough hydrogels. Chem. Eng. J..

[109] GilaVilchez C., RodriguezArco L., MañasTorres M.C., Álvarez de Cienfuegos L., LopezLopez M.T. (2022). Self-assembly in magnetic supramolecular hydrogels. Curr. Opin. Colloid Interface Sci..

[110] Sontakke V.A., Yokobayashi Y. (2022). Programmable Macroscopic Self-Assembly of DNA-Decorated Hydrogels. J. Am. Chem. Soc..

[111] Wachendörfer M., Schräder P., Buhl E.M., Palkowitz A.L., Ben Messaoud G., Richtering W., Fischer H. (2022). A defined heat pretreatment of gelatin enables control of hydrolytic stability, stiffness, and microstructural architecture of fibrin–gelatin hydrogel blends. Biomater. Sci..

[112] Le-The H., Tibbe M., Loessberg-Zahl J., Palma do Carmo M., van der Helm M., Bomer J., van den Berg A., Leferink A., Segerink L., Eijkel J. (2018). Large-scale fabrication of free-standing and sub-μm PDMS through-hole membranes. Nanoscale.

[113] Lee M.H., Huntington M.D., Zhou W., Yang J.-C., Odom T.W. (2011). Programmable Soft Lithography: Solvent-Assisted Nanoscale Embossing. Nano Lett..

[114] Quirós-Solano W.F., Gaio N., Stassen O.M.J.A., Arik Y.B., Silvestri C., Van Engeland N.C.A., Van der Meer A., Passier R., Sahlgren C.M., Bouten C.V.C. (2018). Microfabricated tuneable and transferable porous PDMS membranes for Organs-on-Chips. Sci. Rep..

[115] Apel P. (2001). Track etching technique in membrane technology. Radiat. Meas..

[116] Zargar R., Nourmohammadi J., Amoabediny G. (2016). Preparation, characterization, and silanization of 3D microporous PDMS structure with properly sized pores for endothelial cell culture. Biotechnol. Appl. Biochem..

[117] Qi L., Ruck C., Spychalski G., King B., Wu B., Zhao Y. (2018). Writing Wrinkles on Poly(dimethylsiloxane) (PDMS) by Surface Oxidation with a CO2 Laser Engraver. ACS Appl. Mater. Interfaces.

[118] Yong J., Chen F., Huo J., Fang Y., Yang Q., Zhang J., Hou X. (2018). Femtosecond laser induced underwater superaerophilic and superaerophobic PDMS sheets with through microholes for selective passage of air bubbles and further collection of underwater gas. Nanoscale.

[119] Duan S., Yang K., Wang Z., Chen M., Zhang L., Zhang H., Li C. (2016). Fabrication of Highly Stretchable Conductors Based on 3D Printed Porous Poly(dimethylsiloxane) and Conductive Carbon Nanotubes/Graphene Network. ACS Appl. Mater. Interfaces.

[120] Mathur A., Roy S.S., Tweedie M., Mukhopadhyay S., Mitra S.K., McLaughlin J.A. (2009). Characterisation of PMMA microfluidic channels and devices fabricated by hot embossing and sealed by direct bonding. Curr. Appl. Phys..

[121] Klank H., Kutter J.P., Geschke O. (2002). CO_2_-laser micromachining and back-end processing for rapid production of PMMA-based microfluidic systems. Lab A Chip.

[122] Sedghamiz E., Liu M., Wenzel W. (2022). Challenges and limits of mechanical stability in 3D direct laser writing. Nat. Commun..

[123] Ma X., Li R., Jin Z., Fan Y., Zhou X., Zhang Y. (2020). Injection molding and characterization of PMMA-based microfluidic devices. Microsyst. Technol..

[124] Martinez A.W., Phillips S.T., Butte M.J., Whitesides G.M. (2007). Patterned Paper as a Platform for Inexpensive, Low-Volume, Portable Bioassays. Angew. Chem..

[125] Zhu L., Mei X., Peng Z., Liu J., Yang J., Li Y. (2023). A rotating paper-based microfluidic sensor array combining Michael acceptors and carbon quantum dots for discrimination of biothiols. Chem. Eng. J..

[126] Zhang Y., Khan A.K., See D., Ying J.Y. (2023). Enhancing Protein Adsorption for Improved Lateral Flow Assay on Cellulose Paper by Depleting Inert Additive Films Using Reactive Plasma. ACS Appl. Mater. Interfaces.

[127] Kalish B., Tan M.K., Tsutsui H. (2020). Modifying Wicking Speeds in Paper-Based Microfluidic Devices by Laser-Etching. Micromachines.

[128] Shin J.H., Park J., Park J.-K. (2017). Organic Solvent and Surfactant Resistant Paper-Fluidic Devices Fabricated by One-Step Embossing of Nonwoven Polypropylene Sheet. Micromachines.

[129] Guo M., Wang Y., Gao B., He B. (2021). Shark Tooth-Inspired Microneedle Dressing for Intelligent Wound Management. ACS Nano.

[130] Li X., Li X., Yang J., Lin J., Zhu Y., Xu X., Cui W. (2023). Living and Injectable Porous Hydrogel Microsphere with Paracrine Activity for Cartilage Regeneration. Small.

[131] Wu J., Li G., Ye T., Lu G., Li R., Deng L., Wang L., Cai M., Cui W. (2020). Stem cell-laden injectable hydrogel microspheres for cancellous bone regeneration. Chem. Eng. J..

[132] Ding X., Yu Y., Shang L., Zhao Y. (2022). Histidine-Triggered GO Hybrid Hydrogels for Microfluidic 3D Printing. ACS Nano.

[133] Neiman J.A.S., Raman R., Chan V., Rhoads M.G., Raredon M.S.B., Velazquez J.J., Dyer R.L., Bashir R., Hammond P.T., Griffith L.G. (2015). Photopatterning of hydrogel scaffolds coupled to filter materials using stereolithography for perfused 3D culture of hepatocytes. Biotechnol. Bioeng..

[134] Nilghaz A., Ballerini D.R., Shen W. (2013). Exploration of microfluidic devices based on multi-filament threads and textiles: A review. Biomicrofluidics.

[135] Agustini D., Caetano F.R., Quero R.F., Fracassi da Silva J.A., Bergamini M.F., Marcolino-Junior L.H., de Jesus D.P. (2021). Microfluidic devices based on textile threads for analytical applications: State of the art and prospects. Anal. Methods.

[136] Feng D., Weng D., Wang J. (2019). Interfacial tension gradient driven self-assembly of binary colloidal particles for fabrication of superhydrophobic porous films. J. Colloid Interface Sci..

[137] He B., He J., Bi E., Zou H., Liu T., Liu Z. (2022). Transport and retention of nano emulsified vegetable oil in porous media: Effect of pore straining, roughness wedging, and interfacial effects. J. Environ. Manag..

[138] Stoecklein D., Di Carlo D. (2019). Nonlinear Microfluidics. Anal. Chem..

[139] Youngren G.K., Acrivos A. (2006). Stokes flow past a particle of arbitrary shape: A numerical method of solution. J. Fluid Mech..

[140] Gou Y., Jia Y., Wang P., Sun C. (2018). Progress of Inertial Microfluidics in Principle and Application. Sensors.

[141] Segre G., Silberberg A. (1961). Radial particle displacements in Poiseuille flow of suspensions. Nature.

[142] Segré G., Silberberg A. (2006). Behaviour of macroscopic rigid spheres in Poiseuille flow Part 1. Determination of local concentration by statistical analysis of particle passages through crossed light beams. J. Fluid Mech..

[143] Dean W.R. (2009). XVI. Note on the motion of fluid in a curved pipe. Lond. Edinb. Dublin Philos. Mag. J. Sci..

[144] Dean W.R. (2009). LXXII. The stream-line motion of fluid in a curved pipe (Second paper). Lond. Edinb. Dublin Philos. Mag. J. Sci..

[145] Dinler A., Okumus I. (2018). Inertial particle separation in curved networks: A numerical study. Chem. Eng. Sci..

[146] Gossett D.R., Carlo D.D. (2009). Particle focusing mechanisms in curving confined flows. Anal. Chem..

[147] Sheng P., Wen W. (2012). Electrorheological Fluids: Mechanisms, Dynamics, and Microfluidics Applications. Annu. Rev. Fluid Mech..

[148] Tao R., Sun J.M. (1991). Three-dimensional structure of induced electrorheological solid. Phys. Rev. Lett..

[149] Yethiraj A., van Blaaderen A. (2003). A colloidal model system with an interaction tunable from hard sphere to soft and dipolar. Nature.

[150] Hynninen A.P., Dijkstra M. (2005). Phase diagram of dipolar hard and soft spheres: Manipulation of colloidal crystal structures by an external field. Phys. Rev. Lett..

[151] Bergman D.J., Stroud D. (1992). Physical properties of macroscopically inhomogeneous media. Solid State Physics.

[152] Ma H., Wen W., Tam W.Y., Sheng P. (1996). Frequency dependent electrorheological properties: Origin and bounds. Phys. Rev. Lett..

[153] Tam W.Y., Yi G.H., Wen W., Ma H., Loy M.M., Sheng P. (1997). New electrorheological fluid: Theory and experiment. Phys. Rev. Lett..

[154] Ulep T., Zenhausern R., Gonzales A., Knoff D.S., Lengerke Diaz P.A., Castro J.E., Yoon J. (2020). Smartphone based on-chip fluorescence imaging and capillary flow velocity measurement for detecting ROR1+ cancer cells from buffy coat blood samples on dual-layer paper microfluidic chip. Biosens. Bioelectron..

[155] Washburn E.W. (1921). The Dynamics of Capillary Flow. Phys. Rev..

[156] Mendez S., Fenton E.M., Gallegos G.R., Petsev D.N., Sibbett S.S., Stone H.A., Zhang Y., Lopez G.P. (2010). Imbibition in porous membranes of complex shape: Quasi-stationary flow in thin rectangular segments. Langmuir.

[157] Kar S., Das S.S., Laha S., Chakraborty S. (2020). Microfluidics on Porous Substrates Mediated by Capillarity-Driven Transport. Ind. Eng. Chem. Res..

[158] Buser J.R., Byrnes S.A., Anderson C.E., Howell A.J., Kauffman P.C., Bishop J.D., Wheeler M.H., Kumar S., Yager P. (2019). Understanding partial saturation in paper microfluidics enables alternative device architectures. Anal. Methods.

[159] Darcy H., Dalmont V. (1856). Les Fontaines Publiques de la Ville de Dijon: Exposition et Application des Principes à Suivre et des Formules à Employer Dans les Questions de Distribution D’eau… Un Appendice Relatif aux Fournitures D’eau de Plusieurs Villes au Filtrage des Eaux.

[160] Alcocer F., Kumar V., Singh P. (1999). Permeability of periodic porous media. Phys. Rev. E.

[161] Alcocer F., Singh P. (2002). Permeability of periodic arrays of cylinders for viscoelastic flows. Phys. Fluids.

[162] Chang S., Seo J., Hong S., Lee D.-G., Kim W. (2018). Dynamics of liquid imbibition through paper with intra-fibre pores. J. Fluid Mech..

[163] Cao R., Pan Z., Tang H., Wu J., Tian J., Nilghaz A., Li M. (2020). Understanding the coffee-ring effect of red blood cells for engineering paper-based blood analysis devices. Chem. Eng. J..

[164] Park S., Zhang Y., Lin S., Wang T.-H., Yang S. (2011). Advances in microfluidic PCR for point-of-care infectious disease diagnostics. Biotechnol. Adv..

[165] Nasseri B., Soleimani N., Rabiee N., Kalbasi A., Karimi M., Hamblin M.R. (2018). Point-of-care microfluidic devices for pathogen detection. Biosens. Bioelectron..

[166] Hassan U., Ghonge T., Reddy B., Patel M., Rappleye M., Taneja I., Tanna A., Healey R., Manusry N., Price Z. (2017). A point-of-care microfluidic biochip for quantification of CD64 expression from whole blood for sepsis stratification. Nat. Commun..

[167] Neoh K.H., Hassan A.A., Chen A., Sun Y., Liu P., Xu K., Wong A.S.T., Han R.P.S. (2018). Rethinking liquid biopsy: Microfluidic assays for mobile tumor cells in human body fluids. Biomaterials.

[168] KékedyNagy L., Perry J.M., Little S.R., Llorens O.Y., Shih S.C.C. (2023). An electrochemical aptasensor for Δ9-tetrahydrocannabinol detection in saliva on a microfluidic platform. Biosens. Bioelectron..

[169] Hao Z., Chen H., Shi X., Tan W., Zhu G. (2021). Fabrication for paper-based microfluidic analytical devices and saliva analysis application. Microfluid. Nanofluidics.

[170] Akarapipad P., Kaarj K., Breshears L.E., Sosnowski K., Baker J., Nguyen B.T., Eades C., Uhrlaub J.L., Quirk G., NikolichŽugich J. (2022). Smartphone-based sensitive detection of SARS-CoV-2 from saline gargle samples via flow profile analysis on a paper microfluidic chip. Biosens. Bioelectron..

[171] Mani V., Beduk T., Khushaim W., Ceylan A.E., Timur S., Wolfbeis O.S., Salama K.N. (2021). Electrochemical sensors targeting salivary biomarkers: A comprehensive review. TrAC Trends Anal. Chem..

[172] Viswanath B., Choi C.S., Lee K., Kim S. (2017). Recent trends in the development of diagnostic tools for diabetes mellitus using patient saliva. TrAC Trends Anal. Chem..

[173] Chakraborty P., Dhar S., Deka N., Debnath K., Mondal S.P. (2020). Non-enzymatic salivary glucose detection using porous CuO nanostructures. Sens. Actuators B Chem..

[174] Mao X., Zhang C. (2022). A microfluidic cloth-based photoelectrochemical analytical device for the detection of glucose in saliva. Talanta.

[175] Zhu J., Liu S., Hu Z., Zhang X., Yi N., Tang K., Dexheimer M.G., Lian X., Wang Q., Yang J. (2021). Laser-induced graphene non-enzymatic glucose sensors for on-body measurements. Biosens. Bioelectron..

[176] Xu J., Xu K., Han Y., Wang D., Li X., Hu T., Yi H., Ni Z. (2020). A 3D porous graphene aerogel@ GOx based microfluidic biosensor for electrochemical glucose detection. Analyst.

[177] Vinoth R., Sangavi P., Nakagawa T., Jayaraman M., Mohan A.M.V. (2023). All-in-one microfluidic device with an integrated porous filtration membrane for on-site detection of multiple salivary biomarkers. Sens. Actuators B Chem..

[178] Lin Y., Bariya M., Nyein H.Y.Y., Kivimäki L., Uusitalo S., Jansson E., Ji W., Yuan Z., Happonen T., Liedert C. (2019). Porous Enzymatic Membrane for Nanotextured Glucose Sweat Sensors with High Stability toward Reliable Noninvasive Health Monitoring. Adv. Funct. Mater..

[179] Jia Y., Sun H., Li X., Sun D., Hu T., Xiang N., Ni Z. (2018). Based graphene oxide biosensor coupled with smartphone for the quantification of glucose in oral fluid. Biomed. Microdevices.

[180] Zhang X., Song Y., Fang F., Wu Z. (2018). Sensitive paper-based analytical device for fast colorimetric detection of nitrite with smartphone. Anal. Bioanal. Chem..

[181] Devarakonda S., Singh R., Bhardwaj J., Jang J. (2017). Cost-Effective and Handmade Paper-Based Immunosensing Device for Electrochemical Detection of Influenza Virus. Sensors.

[182] Lewińska I., CapitánVallvey L.F., Erenas M.M. (2023). Thread-based microfluidic sensor for lithium monitoring in saliva. Talanta.

[183] Baker L.B., Seib M.S., Barnes K.A., Brown S.D., King M.A., De Chavez P.J.D., Qu S., Archer J., Wolfe A.S., Stofan J.R. (2022). Skin-Interfaced Microfluidic System with Machine Learning-Enabled Image Processing of Sweat Biomarkers in Remote Settings. Adv. Mater. Technol..

[184] Hong X., Wu H., Wang C., Zhang X., Wei C., Xu Z., Chen D., Huang X. (2022). Hybrid Janus Membrane with Dual-Asymmetry Integration of Wettability and Conductivity for Ultra-Low-Volume Sweat Sensing. ACS Appl. Mater. Interfaces.

[185] Li T., Liang B., Ye Z., Zhang L., Xu S., Tu T., Zhang Y., Cai Y., Zhang B., Fang L. (2022). An integrated and conductive hydrogel-paper patch for simultaneous sensing of Chemical–Electrophysiological signals. Biosens. Bioelectron..

[186] Mogera U., Guo H., Namkoong M., Rahman M.S., Nguyen T., Tian L. (2022). Wearable plasmonic paper–based microfluidics for continuous sweat analysis. Sci. Adv..

[187] Robinson S., Robinson A.H. (1954). Chemical composition of sweat. Physiol. Rev..

[188] TaghizadehBehbahani M., Hemmateenejad B., Shamsipur M., Tavassoli A. (2019). A paper-based length of stain analytical device for naked eye (readout-free) detection of cystic fibrosis. Anal. Chim. Acta.

[189] Wang Z., Dong S., Gui M., Asif M., Wang W., Wang F., Liu H. (2018). Graphene paper supported MoS(2) nanocrystals monolayer with Cu submicron-buds: High-performance flexible platform for sensing in sweat. Anal. Biochem..

[190] Torul H., Çiftçi H., Çetin D., Suludere Z., Boyacı I.H., Tamer U. (2015). Paper membrane-based SERS platform for the determination of glucose in blood samples. Anal. Bioanal. Chem..

[191] Li M., Wang L., Liu R., Li J., Zhang Q., Shi G., Li Y., Hou C., Wang H. (2021). A highly integrated sensing paper for wearable electrochemical sweat analysis. Biosens. Bioelectron..

[192] Bagheri N., Mazzaracchio V., Cinti S., Colozza N., Di Natale C., Netti P.A., Saraji M., Roggero S., Moscone D., Arduini F. (2021). Electroanalytical Sensor Based on Gold-Nanoparticle-Decorated Paper for Sensitive Detection of Copper Ions in Sweat and Serum. Anal. Chem..

[193] Fiore L., Mazzaracchio V., Serani A., Fabiani G., Fabiani L., Volpe G., Moscone D., Bianco G.M., Occhiuzzi C., Marrocco G. (2023). Microfluidic paper-based wearable electrochemical biosensor for reliable cortisol detection in sweat. Sens. Actuators B Chem..

[194] Weng X., Fu Z., Zhang C., Jiang W., Jiang H. (2022). A Portable 3D Microfluidic Origami Biosensor for Cortisol Detection in Human Sweat. Anal. Chem..

[195] Singh A., Hazarika A., Dutta L., Bhuyan A., Bhuyan M. (2022). A fully handwritten-on-paper copper nanoparticle ink-based electroanalytical sweat glucose biosensor fabricated using dual-step pencil and pen approach. Anal. Chim. Acta.

[196] Fabiani L., Mazzaracchio V., Moscone D., Fillo S., De Santis R., Monte A., Amatore D., Lista F., Arduini F. (2022). Paper-based immunoassay based on 96-well wax-printed paper plate combined with magnetic beads and colorimetric smartphone-assisted measure for reliable detection of SARS-CoV-2 in saliva. Biosens. Bioelectron..

[197] Moon J., Del Caño R., Moonla C., Sakdaphetsiri K., Saha T., Francine Mendes L., Yin L., Chang A., Seker S., Wang J. (2022). Self-Testing of Ketone Bodies, along with Glucose, Using Touch-Based Sweat Analysis. ACS Sens..

[198] Gunatilake U.B., GarciaRey S., Ojeda E., BasabeDesmonts L., BenitoLopez F. (2021). TiO_2_ Nanotubes Alginate Hydrogel Scaffold for Rapid Sensing of Sweat Biomarkers: Lactate and Glucose. ACS Appl. Mater. Interfaces.

[199] Guzman J.M.C.C., Hsu S., Chuang H. (2020). Colorimetric Diagnostic Capillary Enabled by Size Sieving in a Porous Hydrogel. Biosensors.

[200] Xu Z., Song J., Liu B., Lv S., Gao F., Luo X., Wang P. (2021). A conducting polymer PEDOT:PSS hydrogel based wearable sensor for accurate uric acid detection in human sweat. Sens. Actuators B Chem..

[201] Siripongpreda T., Somchob B., Rodthongkum N., Hoven V.P. (2021). Bacterial cellulose-based re-swellable hydrogel: Facile preparation and its potential application as colorimetric sensor of sweat pH and glucose. Carbohydr. Polym..

[202] Yeung K.K., Li J., Huang T., Hosseini I.I., Al Mahdi R., Alam M.M., Sun H., Mahshid S., Yang J., Ye T.T. (2022). Utilizing Gradient Porous Graphene Substrate as the Solid-Contact Layer To Enhance Wearable Electrochemical Sweat Sensor Sensitivity. Nano Lett..

[203] Yoon H., Nah J., Kim H., Ko S., Sharifuzzaman M., Barman S.C., Xuan X., Kim J., Park J.Y. (2020). A chemically modified laser-induced porous graphene based flexible and ultrasensitive electrochemical biosensor for sweat glucose detection. Sens. Actuators B Chem..

[204] Wang L., Xu T., Fan C., Zhang X. (2021). Wearable strain sensor for real-time sweat volume monitoring. iScience.

[205] Saha T., Fang J., Mukherjee S., Dickey M.D., Velev O.D. (2021). Wearable Osmotic-Capillary Patch for Prolonged Sweat Harvesting and Sensing. ACS Appl. Mater. Interfaces.

[206] Liu Q., Shi W., Tian L., Su M., Jiang M., Li J., Gu H., Yu C. (2021). Preparation of nanostructured PDMS film as flexible immunosensor for cortisol analysis in human sweat. Anal. Chim. Acta.

[207] Li B., Wu X., Shi C., Dai Y., Zhang J., Liu W., Wu C., Zhang Y., Huang X., Zeng W. (2023). Flexible enzymatic biosensor based on graphene sponge for glucose detection in human sweat. Surf. Interfaces.

[208] Xuan X., Kim J.Y., Hui X., Das P.S., Yoon H.S., Park J. (2018). A highly stretchable and conductive 3D porous graphene metal nanocomposite based electrochemical-physiological hybrid biosensor. Biosens. Bioelectron..

[209] Kil M.S., Kim S.J., Park H.J., Yoon J.H., Jeong J., Choi B.G. (2022). Highly Stretchable Sensor Based on Fluid Dynamics-Assisted Graphene Inks for Real-Time Monitoring of Sweat. ACS Appl. Mater. Interfaces.

[210] Liu X., Zhang W., Lin Z., Meng Z., Shi C., Xu Z., Yang L., Liu X.Y. (2021). Coupling of Silk Fibroin Nanofibrils Enzymatic Membrane with Ultra-Thin PtNPs/Graphene Film to Acquire Long and Stable On-Skin Sweat Glucose and Lactate Sensing. Small Methods.

[211] Xu M., Zhu Y., Gao S., Zhang Z., Gu Y., Liu X. (2021). Reduced Graphene Oxide-Coated Silica Nanospheres as Flexible Enzymatic Biosensors for Detection of Glucose in Sweat. ACS Appl. Nano Mater..

[212] Poletti F., Zanfrognini B., Favaretto L., Quintano V., Sun J., Treossi E., Melucci M., Palermo V., Zanardi C. (2021). Continuous capillary-flow sensing of glucose and lactate in sweat with an electrochemical sensor based on functionalized graphene oxide. Sens. Actuators B Chem..

[213] Park J.S., Choi J.S., Han D.K. (2022). Platinum nanozyme-hydrogel composite (PtNZHG)-impregnated cascade sensing system for one-step glucose detection in serum, urine, and saliva. Sens. Actuators B Chem..

[214] Elancheziyan M., Prakasham K., Eswaran M., Duraisamy M., Ganesan S., Lee S.L., Ponnusamy V.K. (2023). Eco-friendly fabrication of nonenzymatic electrochemical sensor based on cobalt/polymelamine/nitrogen-doped graphitic-porous carbon nanohybrid material for glucose monitoring in human blood. Environ. Res..

[215] Yao T., Dong G., Qian S., Cui Y., Chen X., Tan T., Li L. (2022). Persistent luminescence nanoparticles/hierarchical porous ZIF-8 nanohybrids for autoluminescence-free detection of dopamine. Sens. Actuators B Chem..

[216] Marques M.P.C., Szita N. (2017). Bioprocess microfluidics: Applying microfluidic devices for bioprocessing. Curr. Opin. Chem. Eng..

[217] Charmet J., Arosio P., Knowles T.P.J. (2018). Microfluidics for Protein Biophysics. J. Mol. Biol..

[218] Zhao X., Liu Y., Yu Y., Huang Q., Ji W., Li J., Zhao Y. (2018). Hierarchically porous composite microparticles from microfluidics for controllable drug delivery. Nanoscale.

[219] Martins J.P., Liu D., Fontana F., Ferreira M.P.A., Correia A., Valentino S., Kemell M., Moslova K., Mäkilä E., Salonen J. (2018). Microfluidic Nanoassembly of Bioengineered Chitosan-Modified FcRn-Targeted Porous Silicon Nanoparticles @ Hypromellose Acetate Succinate for Oral Delivery of Antidiabetic Peptides. ACS Appl. Mater. Interfaces.

[220] Zhang H., Liu D., Shahbazi M., Mäkilä E., HerranzBlanco B., Salonen J., Hirvonen J., Santos H.A. (2014). Fabrication of a Multifunctional Nano-in-micro Drug Delivery Platform by Microfluidic Templated Encapsulation of Porous Silicon in Polymer Matrix. Adv. Mater..

[221] Zhang H., Liu Y., Wang J., Shao C., Zhao Y. (2019). Tofu-inspired microcarriers from droplet microfluidics for drug delivery. Sci. China Chem..

[222] Sanjay S.T., Zhou W., Dou M., Tavakoli H., Ma L., Xu F., Li X. (2018). Recent advances of controlled drug delivery using microfluidic platforms. Adv. Drug Deliv. Rev..

[223] Chen R., Wulff J.E., Moffitt M.G. (2018). Microfluidic Processing Approach to Controlling Drug Delivery Properties of Curcumin-Loaded Block Copolymer Nanoparticles. Mol. Pharm..

[224] Surawathanawises K., Wiedorn V., Cheng X. (2017). Micropatterned macroporous structures in microfluidic devices for viral separation from whole blood. Analyst.

[225] Yin J., Mou L., Yang M., Zou W., Du C., Zhang W., Jiang X. (2019). Highly efficient capture of circulating tumor cells with low background signals by using pyramidal microcavity array. Anal Chim Acta.

[226] Yao X., Choudhury A.D., Yamanaka Y.J., Adalsteinsson V.A., Gierahn T.M., Williamson C.A., Lamb C.R., Taplin M.E., Nakabayashi M., Chabot M.S. (2014). Functional analysis of single cells identifies a rare subset of circulating tumor cells with malignant traits. Integr. Biol..

[227] Chen X., Cui D., Liu C., Li H., Chen J. (2007). Continuous flow microfluidic device for cell separation, cell lysis and DNA purification. Anal. Chim. Acta.

[228] Mitra S., Chakraborty A.J., Tareq A.M., Emran T.B., Nainu F., Khusro A., Idris A.M., Khandaker M.U., Osman H., Alhumaydhi F.A. (2022). Impact of heavy metals on the environment and human health: Novel therapeutic insights to counter the toxicity. J. King Saud Univ. Sci..

[229] Meng Q. (2010). Three-dimensional culture of hepatocytes for prediction of drug-induced hepatotoxicity. Expert Opin. Drug Metab. Toxicol..

[230] Su Y., Hu X., Kang Y., Zhang C., Cheng Y.Y., Jiao Z., Nie Y., Song K. (2023). Curcumin nanoparticles combined with 3D printed bionic tumor models for breast cancer treatment. Biofabrication.

[231] Kappes M., Friedrich B., Pfister F., Huber C., Friedrich R.P., Stein R., Braun C., Band J., Schreiber E., Alexiou C. (2022). Superparamagnetic Iron Oxide Nanoparticles for Targeted Cell Seeding: Magnetic Patterning and Magnetic 3D Cell Culture. Adv. Funct. Mater..

[232] Yu J.Z., Korkmaz E., Berg M.I., LeDuc P.R., Ozdoganlar O.B. (2017). Biomimetic scaffolds with three-dimensional undulated microtopographies. Biomaterials.

[233] Li Q., Hatakeyama M., Kitaoka T. (2022). Bioadaptive Porous 3D Scaffolds Comprising Cellulose and Chitosan Nanofibers Constructed by Pickering Emulsion Templating. Adv. Funct. Mater..

[234] Li P., Chen J., Chen Y., Song S., Huang X., Yang Y., Li Y., Tong Y., Xie Y., Li J. (2023). Construction of Exosome SORL1 Detection Platform Based on 3D Porous Microfluidic Chip and its Application in Early Diagnosis of Colorectal Cancer. Small.

